# On the Optimality of Interference Decoding Schemes for *K*-User Gaussian Interference Channels †

**DOI:** 10.3390/e21111053

**Published:** 2019-10-28

**Authors:** Ragini Chaluvadi, Madhuri Bolli, Srikrishna Bhashyam

**Affiliations:** 1 Department of Electrical Engineering, Indian Institute of Technology Madras, Chennai 600036, India; chaluvadiragini@gmail.com; 2 Qualcomm India Pvt Limited, Hyderabad, Telangana 500081, India; madhuri.bolli@gmail.com

**Keywords:** Gaussian Interference Channel, Han-Kobayashi scheme, treating interference as noise (TIN)

## Abstract

The sum capacity of the general *K*-user Gaussian Interference Channel (GIC) is known only when the channel coefficients are such that treating interference as noise (TIN) is optimal. The Han-Kobayashi (HK) scheme is an extensively studied coding scheme for the *K*-user interference channel (IC). Simple HK schemes are HK schemes with Gaussian signaling, no time sharing and no private-common power splitting. The class of simple HK (S-HK) schemes includes the TIN scheme and schemes that involve various levels of interference decoding and cancellation at each receiver. For the 2-user GIC, simple HK schemes are sufficient to achieve all known sum capacity results—sum capacity under mixed, strong and noisy interference conditions. We derive channel conditions under which simple HK schemes achieve sum capacity for general *K*-user Gaussian ICs. For the *K*-user GIC, these results generalize existing sum capacity results for the TIN scheme to the class of simple HK schemes.

## 1. Introduction

Wireless cellular networks have evolved significantly in terms of both channel-adaptive transmission and interference management. Early cellular systems were based on the *interference avoidance* approach and relied on static resource allocation. However, current cellular systems allocate resources dynamically based on short-term channel state feedback. Interference decoding and cancellation are also implementable in today’s systems. The *K*-user Gaussian Interference Channel (GIC) models a wireless network with *K* transmit-receive pairs. The optimal transmission scheme for the *K*-user GIC depends on the channel coefficients. Simultaneous channel-aware adaptation of multiple transmit-receive pairs requires a good understanding of the optimal scheme for each channel condition.

The capacity region and sum capacity of the general *K*-user Gaussian Interference Channel (GIC) are not known. The 2-user GIC is the most well understood special case [1,2,3,4,5,6,7]. The capacity region of the 2-user GIC under strong interference conditions was obtained in References [1,2]. The sum capacity when the interference can be treated as noise was obtained in References [3,4,5,6]. The sum capacity under mixed interference conditions was obtained in Reference [6]. The capacity region of the 2-user GIC within one bit was derived in Reference [7] using suitably chosen Han-Kobayashi (HK) schemes [8].

For the general *K*-user GIC, the channel conditions under which Treating Interference as Noise (TIN) achieves sum capacity were obtained in References [5] (Thm. 3) and [9] (Thm. 9). The sum capacity of some *partially connectedK* user GICs were derived in Reference [10,11,12,13] under some channel conditions. *Z*-like GICs, where the channel matrix is upper triangular with a specific structure, were studied in Reference [10], cascade GIC was studied in Reference [11] and many-to-one and one-to-many GICs were studied in References [12,13]. Some new outer bounds on the capacity of the *K*-user GIC were recently derived in Reference [14]. *Simple* HK (S-HK) schemes with Gaussian signalling, no timesharing and no common-private power splitting, achieve sum capacity under the channel conditions obtained in References [9,10,11,12,13]. S-HK schemes include the simple and practical TIN scheme and schemes that involve various levels of interference decoding and cancellation at each receiver as special cases. For the 2-user GIC, S-HK schemes are sufficient to achieve all known sum capacity results. For the *K*-user GIC, we will generalize the sum capacity optimality results for the TIN scheme in References [5,9], to S-HK schemes.

There are some other related results for the *symmetric* GIC or for GICs where the channel coefficients satisfy some equality conditions [15,16], respectively. For a *K*-user interference channel, the sum capacity under a strong interference condition was obtained in Reference [16] under conditions that include some equality conditions on the channel coefficients. Equality conditions cannot be satisfied if the channel coefficients come from continuous distributions. The symmetric *K*-user GIC and many-to-one GIC have been considered in References [15,17], respectively. Unlike the other results discussed above based on S-HK schemes, in References [15,17], lattice coding and interference alignment are used to obtain the sum capacity when the interference is very strong. For the general asymmetric GIC, only approximate sum capacity and degrees of freedom results have been obtained using interference alignment. Other structured codes like coset codes have also been studied in Reference [18] to show achievable sum rates better than those achieved by HK schemes for some 3-user interference channels. Interference alignment and structured codes are useful under channel conditions where HK schemes are not optimal. We identify channel conditions where S-HK schemes are sum capacity optimal for the *K*-user GIC.

In this paper, we generalize the sum capacity optimality results for the TIN scheme in References [5,9] to S-HK schemes. In particular, we derive two sets of channel conditions under which S-HK schemes are sum capacity optimal for general *K*-user GICs. For the first set of channel conditions, we consider schemes where interference is decoded and cancelled before decoding the desired message. For the second set of channel conditions, we consider schemes where the one interference signal is jointly decoded with the message signal at one of the receivers. These two sets of channel conditions provide us new sum capacity results for several channel conditions under which sum capacity was not known earlier. Furthermore, existing results for the sum capacity of the 2-user GIC and some partially connected *K* user GICs in References [11,12,13] can be obtained as special cases of these results. To further understand the significance of the results, we evaluate, using Monte Carlo simulations, the probability that these channel conditions for sum capacity are satisfied for random wireless networks. Three different random network models are considered and we observe that this probability is significant under all three models.

## 2. Channel Model and Simple HK Schemes

The *K*-user GIC in standard form [5] is given by

(1) $$y_{i}=x_{i}+\underset{\begin{array}{c} j=1\\ j\neq i \end{array}}{\sum^{K}}h_{ij}x_{j}+z_{i},\;\forall i\in \left[K\right]≜\left\{1,\cdots ,K\right\},$$

where $x_{i}$ is transmitted by transmitter *i*, $y_{i}$ is received by receiver *i*, $h_{ij}$ is the real channel coefficient from transmitter *j* to receiver *i* and $z_{i}∼N\left(0,1\right)$ is the additive white Gaussian noise at receiver *i*. Let $P_{i}$ denote the transmit power constraint at transmitter *i*. For the 2-user GIC, the HK scheme [8] splits the message at each user into two parts; common and private. The common message is decoded at both the receivers and private part is decoded only at its corresponding receiver. This scheme can be generalized to the *K*-user GIC in several ways [19] (Sec. 6.9). In Reference [20], the message at each user of a *K*-user GIC is split into two—common and private messages. The common message is decoded at all receivers. A more general scheme can split each message into more than two parts and specify the subset of receivers that can decode each part. We consider schemes where there is only one message, but that is decoded by the intended receiver and a subset of the remaining K−1 receivers. Equivalently, these schemes can also be described by specifying the messages that are decoded at each receiver. We call such HK schemes with Gaussian signaling, no timesharing and no common-private power splitting as simple HK schemes. Each S-HK scheme is specified by the sets {I(1),I(2),⋯,I(K)}, I(i)⊆[K]∖{i},∀i. In each such S-HK scheme, at receiver *i*, interference from transmitters j∈I(i) are treated as noise and interference from transmitters j∈D(i)≜{[K]∖{I(i),i}} are decoded. For the TIN scheme, I(i)=[K]∖{i}, ∀i.

## 3. Sum Capacity Results

In this section, we derive two sets of channel conditions for the general *K*-user GIC under which sum capacity is achieved by S-HK schemes. The first set of channel conditions are in Equations (2)–(4) of Theorem 1. The second set of channel conditions are given by Equations (6), (7)–(10) in Theorems 2 and 3, respectively.

In the result in Theorem 1, we consider the strategy of decoding interference from transmitters in D(i) for each *i* before decoding the desired message. For such decoding to be possible, conditions in (4) need to be satisfied. For the optimality of treating the interference from transmitters in I(i) as noise for each *i*, we get conditions (2) and (3). These conditions correspond to the TIN optimality conditions for the modified GIC where all the links corresponding to decoded interference are removed.

**Theorem** **1.** *For the K-user GIC, the S-HK scheme defined by I(i)⊆[K]∖{i},∀i∈[K] achieves sum capacity, if there exist $\rho_{i}\in \left(0,1\right)$, ∀i∈[K], such that the following conditions are satisfied for all i∈[K]*

(2) $$\begin{array}{c} \sum_{j:i\in I(j)}\left[\frac{h_{ji}^{2}}{1+Q_{j}-\rho_{j}^{2}}\right]\leq \frac{1}{P_{i}+{\left(\frac{1+Q_{i}}{\rho_{i}}\right)}^{2}}, \end{array}$$

(3) $$\begin{array}{c} \sum_{j\in I(i)}\frac{h_{ij}^{2}{\left(1+Q_{j}\right)}^{2}}{\rho_{j}^{2}}\leq 1-\rho_{i}^{2}, \end{array}$$

(4) $$\begin{array}{c} \prod_{j\in J}\left(1+\frac{P_{j}}{1+Q_{j}}\right)\leq \left(1+\frac{\sum_{j\in J}h_{ij}^{2}P_{j}}{1+P_{i}+Q_{i}}\right)\forall J\subseteq D\left(i\right), \end{array}$$

*where $Q_{i}=\sum_{j\in I(i)}h_{ij}^{2}P_{j}$, D(i)=[K]∖{i,I(i)}. The sum capacity is*

(5) $$C_{sum}=\underset{i=1}{\sum^{K}}\frac{1}{2}\log\left[1+\frac{P_{i}}{1+Q_{i}}\right].$$

**Proof.**  The detailed proof is given in Appendix A. For the converse, at each receiver i∈[K], we use the genie signal $s_{i}^{n}=\left\{x_{i}^{n}+n_{i}^{n},\;x_{j}^{n},\;j\in D\left(i\right)\right\}$ where $n_{i}^{n}∼N\left(0,\sigma_{i}^{2}I\right)$ and $E\left[n_{i}z_{i}\right]=\rho_{i}\sigma_{i}$, $0<\rho_{i}<1$. Here, for each *i*, we provide signals $x_{j}^{n},\forall j\in D\left(i\right)$ in addition to the genie signal $x_{i}^{n}+n_{i}^{n}$ that is used in Reference [9]. Under (2) and (3), we get the required upper bound following steps similar to the proof in Reference [9] (Theorem 9) but with the above genie signals.

Combining the conditions (2) and (3) for the converse with the conditions (4) for achievability, we get the required result. □

Now, we derive the second set of channel conditions under which S-HK schemes are optimal. We do this in two steps. First, we derive general bounds on the achievable sum rate of S-HK schemes in Theorem 2. Unlike Theorem 1, where the interference is decoded and cancelled before decoding the desired signal, here we determine more general bounds on the achievable sum rate for an S-HK scheme. Then, we show in Theorem 3 that one of the sum rate upperbounds in Theorem 2 is also a sum capacity bound under some channel conditions. Therefore, the channel conditions under which we get a sum capacity result will comprise of (i) the conditions (7)–(10) required to prove the sum capacity upper bound in Theorem 3 and (ii) the conditions (6) under which this sum capacity upperbound is achievable in Theorem 2.

**Theorem** **2.** *For the K-user GIC, the S-HK scheme defined by {I(i)} achieves sum rates S satisfying the following conditions for each l∈[K].*

(6) $$l.S\leq \frac{1}{2}\sum_{i\in [K]}\log\left(1+\frac{\sum_{j\in J_{i}}h_{ij}^{2}P_{j}}{1+Q_{i}}\right)$$

*for each choice of $J_{i}\subseteq \left[K\right]\setminus I\left(i\right)$ such that $\underset{i\in \left[K\right]}{⋃}J_{i}=S_{l}$. Here $S_{l}$ is a multiset containing l copies of each element in [K] and is denoted $S_{l}=\left\{\left(a,l\right):a\in \left[K\right]\right\}$ and $Q_{i}=\sum_{j\in I(i)}h_{ij}^{2}P_{j}$.*

**Proof.**  At each receiver *i*, users [K]∖I(i) form a Gaussian MAC with noise variance $1+Q_{i}$. The achievable rates of each MAC at receiver i∈[K] satisfy

$$\begin{array}{cc} \sum_{j\in J_{i}}R_{j} & \leq \frac{1}{2}\log\left(1+\frac{\sum_{j\in J_{i}}h_{ij}^{2}P_{j}}{1+Q_{i}}\right)\forall J_{i}\subseteq \left[K\right]\setminus I\left(i\right). \end{array}$$

Using Fourier-Motzkin elimination, we get the sum rate bounds in (6). □

The maximum sum rate achievable using an S-HK scheme is determined by the least lower bound for *S* among the bounds in (6). As an example of a bound in the above theorem, consider l=1, $J_{m}=\left\{m,k\right\}$ for some m,k∈[K], $J_{k}=\phi$ and $J_{i}=i$ for i∈[K]∖{m,k}. This gives us the bound on sum rate to be

$$\frac{1}{2}\log\left[1+\frac{P_{m}+h_{mk}^{2}P_{k}}{1+Q_{m}}\right]+\underset{i\neq k,m}{\underset{i=1}{\sum^{K}}}\frac{1}{2}\log\left[1+\frac{P_{i}}{1+Q_{i}}\right].$$

Now, if we can show that one of these inequalities in (6) is also an upper bound on the sum capacity under some conditions, then we get a sum capacity result. In the following theorem, we show that the sum rate bound expression in the example above is a sum capacity upper bound under conditions (7)–(10) (for the choice G(i)=I(i) in the following theorem).

**Theorem** **3.** *Let G(i)⊆[K]∖{i},∀i∈[K] and let there be some m,k∈[K] such that m,k∉G(i), ∀i∈[K]∖{k}. For the K-user GIC, if there exist $\rho_{i}\in \left(0,1\right)$, ∀i∈[K]∖{m} such that the following conditions are satisfied*

(7) $$\begin{array}{cc} \frac{1}{P_{r}+{\left(\frac{1+Q_{r}}{\rho_{r}}\right)}^{2}} & \geq \sum_{\underset{i\neq \{m,k\}}{i:r\in G(i)}}\left[\frac{h_{ir}^{2}}{1+Q_{i}-\rho_{i}^{2}}\right]+\delta_{r}\left[\frac{h_{mr}^{2}}{1+Q_{m}-\rho_{k}^{2}}\right]\forall \;r\in \left[K\right]\setminus \left\{m,k\right\}, \end{array}$$

(8) $$\begin{array}{cc} \rho_{k}h_{mk} & =1+Q_{m} \end{array}$$

(9) $$\begin{array}{cc} \sum_{j\in G(i)}\frac{h_{ij}^{2}{\left(1+Q_{j}\right)}^{2}}{\rho_{j}^{2}} & \leq 1-\rho_{i}^{2},\forall i\in \left[K\right]\setminus \left\{m,k\right\}, \end{array}$$

(10) $$\begin{array}{cc} \sum_{j\in G(m)}\frac{h_{mj}^{2}{\left(1+Q_{j}\right)}^{2}}{\rho_{j}^{2}} & \leq 1-\rho_{k}^{2}, \end{array}$$

*where $\delta_{r}=1$ if r∈G(m) and $\delta_{r}=0$ otherwise and $Q_{i}=\sum_{j\in G(i)}h_{ij}^{2}P_{j}$, then the sum capacity $C_{sum}$ is upper bounded by*

$$\begin{array}{cc} C_{sum} & \leq \frac{1}{2}\log\left[1+\frac{\left(P_{m}+h_{mk}^{2}P_{k}\right)}{1+Q_{m}}\right]+\underset{i\neq k,m}{\underset{i=1}{\sum^{K}}}\frac{1}{2}\log\left[1+\frac{P_{i}}{1+Q_{i}}\right]. \end{array}$$

**Proof.**  The detailed proof is provided in Appendix B. Here, we present a brief outline and highlight some aspects of the proof. First, we consider a modified channel with no interference at receiver *k*. The sum capacity of the original channel is upper bounded by the sum capacity of the modified channel. Then, we derive a genie-aided upper bound for the modified channel using the genie signals $s_{i}^{n}$ at receiver *i* for each i∈[K] as follows:

$$\begin{array}{cc} s_{i}^{n} & =\{x_{i}^{n}+n_{i}^{n},x_{j}^{n},j\in \bar{G}\left(i\right)\},\;\forall i\in \left[K\right]\setminus \left\{m,k\right\}\\ s_{m}^{n} & =\{x_{j}^{n},\;j\in \bar{G}\left(m\right)\setminus k\}\\ s_{k}^{n} & =h_{mk}x_{k}^{n}+\sum_{j\in G(m)}h_{mj}x_{j}^{n}+n_{k}^{n} \end{array}$$

where $\bar{G}\left(i\right)=\left[K\right]\setminus \left\{i,\left\{G\left(i\right)\right\}\right\}$, $n_{i}∼N\left(0,\sigma_{i}^{2}\right)$, $E\left[n_{i}z_{i}\right]=\rho_{i}\sigma_{i}$ and $0<\rho_{i}<1$, for each i∈[K]∖{m} and $\sigma_{k}=1$. This choice of genie is then shown to be useful and smart under conditions (7)–(10) to obtain the upper bound in the theorem statement.

Here are some remarks about this proof.

- The genie signal is different from Theorem 1 for receivers *m* and *k*. The genie at receiver *k* has the interference component at receiver *m* from transmitter *k* and the other transmitters that are treated as noise. This choice ensures that $h\left(s_{k}^{n}\right)=h\left(y_{m}^{n}|s_{m}^{n},x_{m}^{n}\right)$ and helps in cancelling one negative term in the sum capacity upper bound.
- The assumption that m,k∉G(i), ∀i∈[K]∖{k} is used as part of the argument that the genie is useful.
- The first upper bounding step is with a modified channel with no interference at receiver *k*. It is interesting to note that the sum capacity result for the 2-user GIC under mixed interference in Reference [6] also uses the one-sided GIC as the first step and we recover these results as special cases of our result.
- This proof also generalizes the proof for the many-to-one GIC in Reference ([13], Theorem 4) to the general *K* user GIC. □

Some examples of the conditions obtained from Theorems 1–3 are presented in Appendix C.

### Relation with Existing Sum Capacity Results

Applying Theorems 1–3 to the special case of 2-user channels, that is, K=2, we recover all known sum capacity results for the 2-user GIC in References [1,2,3,4,6]. As special cases, the first set of channel conditions in our paper gives the noisy interference result in References [3,4,6], the very strong interference result in Reference [1] and part of the mixed interference result in Reference [6] (Thm. 10) where the interference is decoded before decoding the message. The second set of channel conditions in our paper gives the remaining part of the mixed interference result in Reference [6] (Thm. 10) where the interference is jointly decoded with the desired message and the strong interference result in Reference [2]. The actual list of channel conditions and the corresponding sum capacity can be found in Appendix D.

Applying Theorems 1–3 to the special cases of partially connected Gaussian ICs, we can recover the sum capacity results in References [11,12,13]. We can also get some new results for the *K*-user cyclic and cascade GICs. The results corresponding to the two channel conditions for the cyclic, cascade and many-to-one GICs are presented in Appendix E.

## 4. Numerical Results

In this section, we numerically find the probability that the first set of channel conditions under which S-HK schemes achieve sum capacity, i.e, Equations (2)–(4), are satisfied for three different random wireless network topologies. Theoretical analysis of the probability of the event that the channel conditions (2)–(4) required for the sum capacity result are satisfied is difficult because of the following reasons: (1) There are many conditions that describe the event, (2) Each condition is a complicated function of channel coefficients, (3) The variables $\rho_{1},\rho_{2},\cdots ,\rho_{K}$ in the conditions are not available in closed form. Therefore, we resort to Monte Carlo simulations in this paper.

**Figure 1:**
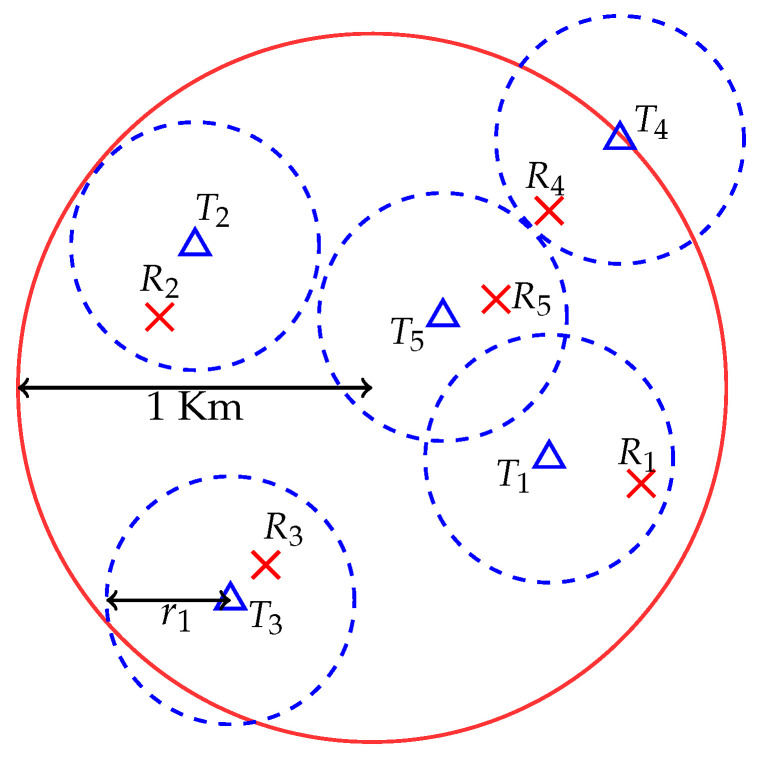
Topology 1 setup where triangles are transmitters and crosses are receivers.

*Topology 1*: In this topology, all *K* transmitters are placed randomly and uniformly in a circular cell of radius 1 km. We assume that each transmitter has a nominal coverage radius of $r_{1}$ m. For each transmitter, we then place its receiver randomly and uniformly in its coverage area. This topology is illustrated in Figure 1 for K=5.

**Figure 2:**
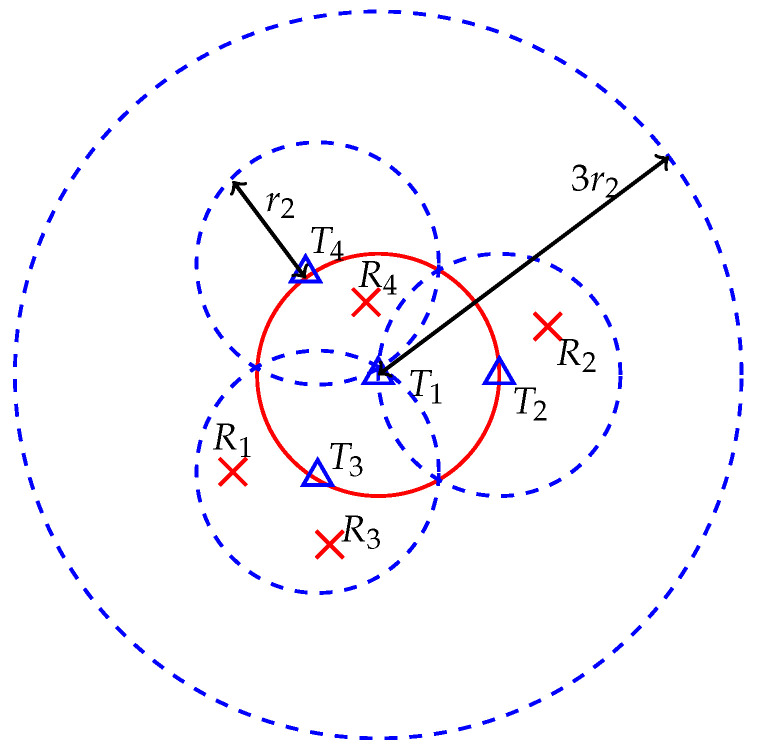
Topology 2 setup where triangles are transmitters and crosses are receivers.

*Topology 2 (Motivated by the one-to-many channel)*: In this topology, the first transmitter is placed at the center of a circle of radius $r_{2}$ m and all the other transmitters are placed equally spaced on the perimeter of this circle. The nominal coverage radius of first transmitter is $3r_{2}$ m and nominal coverage radius of all other transmitters are $r_{2}$ m. For each transmitter, we place its receiver randomly and uniformly in its coverage area. This topology for K=4 is illustrated in Figure 2. In topology 2, the first transmitter has a longer range and, therefore, there is higher probability that its signal at other receivers is strong enough to decode.

**Figure 3:**
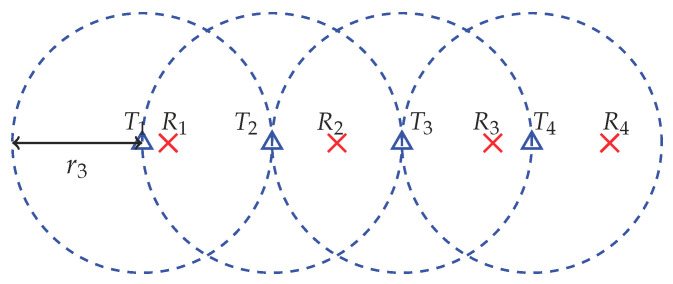
Topology 3 setup where triangles are transmitters and crosses are receivers.

*Topology 3 (Motivated by the cascade channel)*: In this topology, all transmitters are placed equidistantly along a line, with transmitter to transmitter distance $r_{3}$ m. For each transmitter, we place its corresponding receiver randomly and uniformly along the same line towards its right within $r_{3}$ m. We assume that the nominal coverage radius of each transmitter is $r_{3}$ m. This topology for K=4 is illustrated in Figure 3. In topology 3, each receiver usually observes strong interference only from its adjacent transmitter.

For channel fading, we use the Erceg model [21]. We consider two terrain categories, hilly/light tree density (terrain type 1) and hilly/moderate-to-heavy tree density (terrain type 2). The model parameters for the two terrain categories are given in Reference [21] (Table I). We have reproduced the parameter values in Appendix F for completeness. We used an operating frequency of 1.9 GHz, antenna height $h_{b}=50$ m, close-in distance $d_{0}=100$ m. The noise floor is taken as −110 dBm and transmit power at each transmitter is chosen such that the expected value of the SNR at the boundary of their nominal coverage area is 0 dB.

For generating the plots, we consider 1000 realizations of the channel. With topology 1, for every realization we randomly place *K* transmitters inside 1 km circular cell and also randomly place each receiver in its corresponding transmitters coverage area. With topology 2 and topology 3, first we fix the transmitters locations and for every realization we randomly place each receiver in its corresponding transmitter’s coverage area.

**Figure 4:**
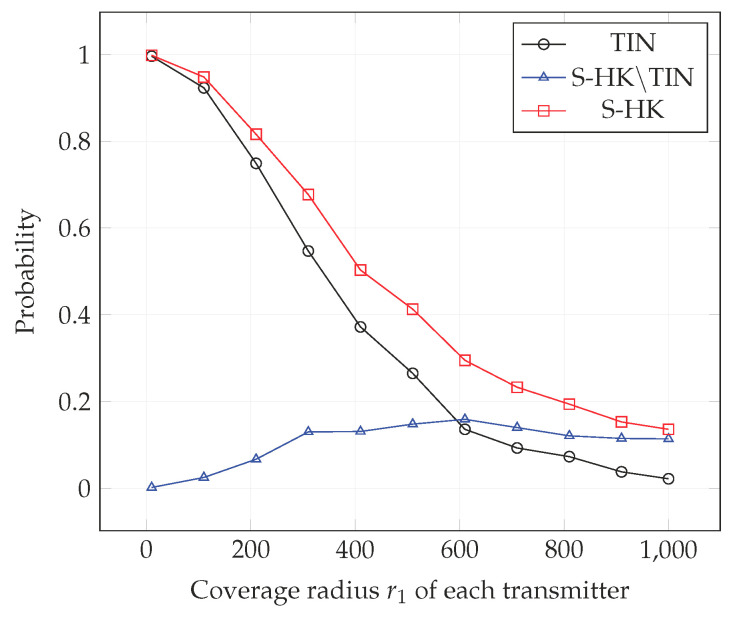
Success probability of conditions (2)–(4) for TIN scheme, S-HK schemes excluding TIN and all S-HK schemes with topology 1, terrain type 1, K=3.

**Figure 5:**
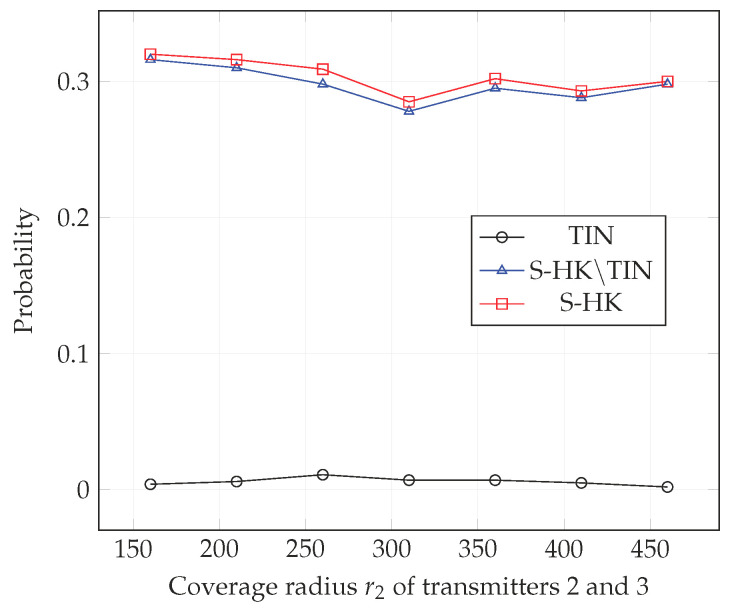
Success probability of conditions (2)–(4) for TIN scheme, S-HK schemes excluding TIN and all S-HK schemes with topology 2, terrain type 1, K=3.

**Figure 6:**
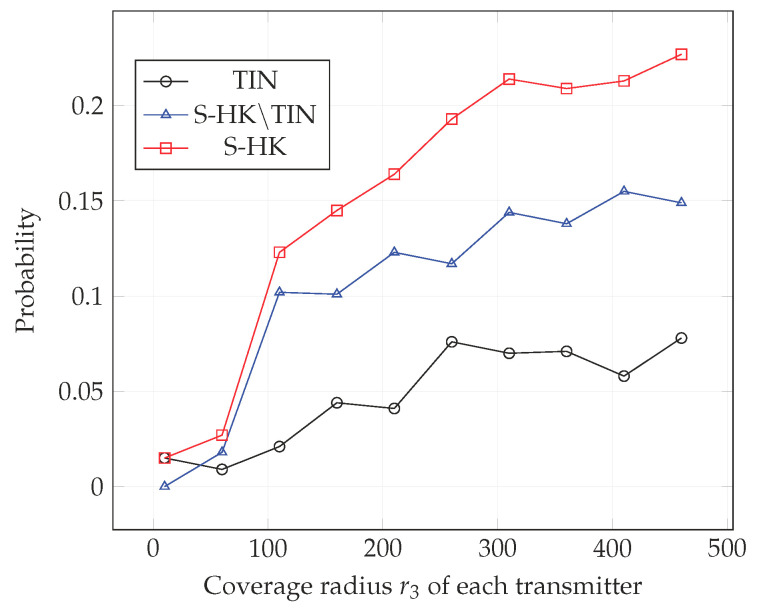
Success probability of conditions (2)–(4) for TIN scheme, S-HK schemes excluding TIN and all S-HK schemes with topology 3, terrain type 1, K=3.

**Figure 7:**
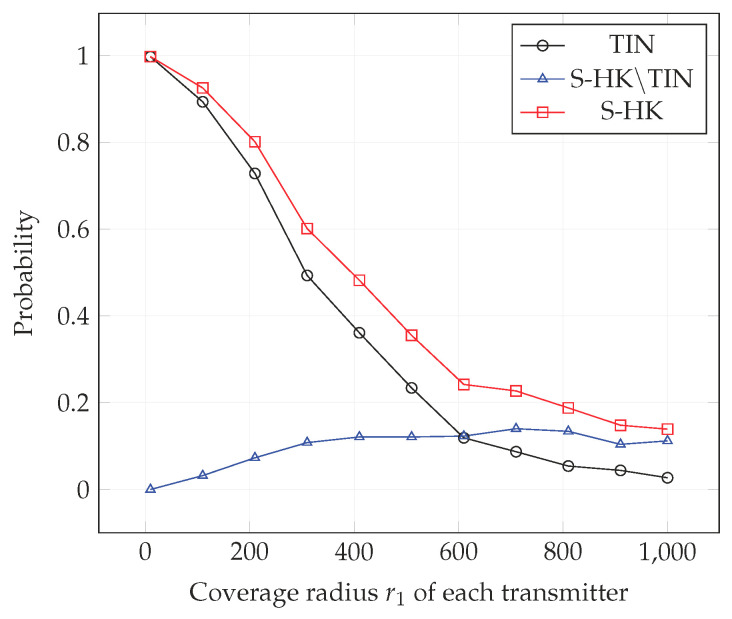
Success probability of conditions (2)–(4) for TIN scheme, S-HK schemes excluding TIN and all S-HK schemes with topology 1, terrain type 2, K=3.

**Figure 8:**
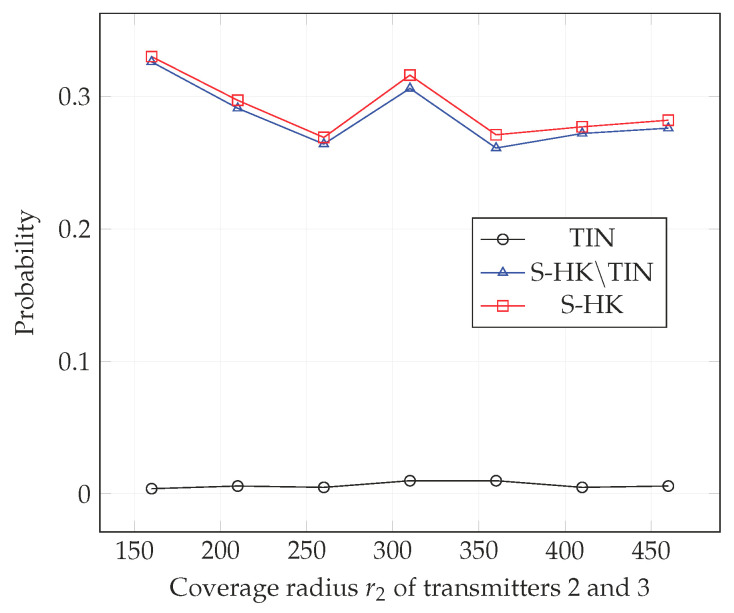
Success probability of conditions (2)–(4) for TIN scheme, S-HK schemes excluding TIN and all S-HK schemes with topology 2, terrain type 2, K=3.

**Figure 9:**
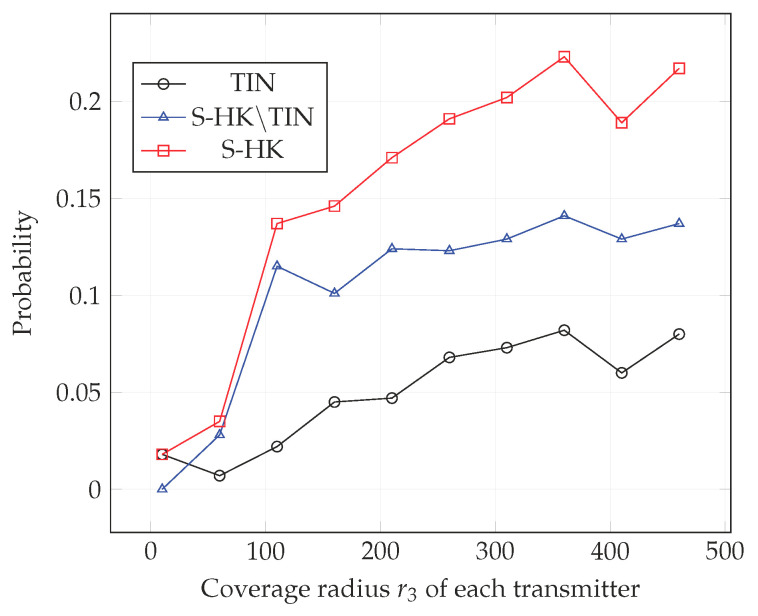
Success probability of conditions (2)–(4) for TIN scheme, S-HK schemes excluding TIN and all S-HK schemes with topology 3, terrain type 2, K=3.

In Figure 4, Figure 5, Figure 6, Figure 7, Figure 8 and Figure 9, we plot the probability that the conditions (2)–(4) are satisfied for (i) TIN scheme, (ii) all S-HK schemes except the TIN scheme (denoted S-HK∖TIN) and (iii) all S-HK schemes. Figure 4, Figure 5 and Figure 6 are plotted for terrain type 1 and Figure 7, Figure 8 and Figure 9 are plotted for terrain type 2. From the Figure 4, Figure 5, Figure 6, Figure 7, Figure 8 and Figure 9, we observe that the probability that the conditions for optimality are satisfied is significant. In Figure 4 and Figure 7 this probability increases with increasing nominal coverage radius $r_{1}$ as expected for S−HK∖TIN. In Figure 5, Figure 6, Figure 8 and Figure 9, S-HK schemes have a much higher probability of being optimal compared to the TIN scheme.

**Figure 10:**
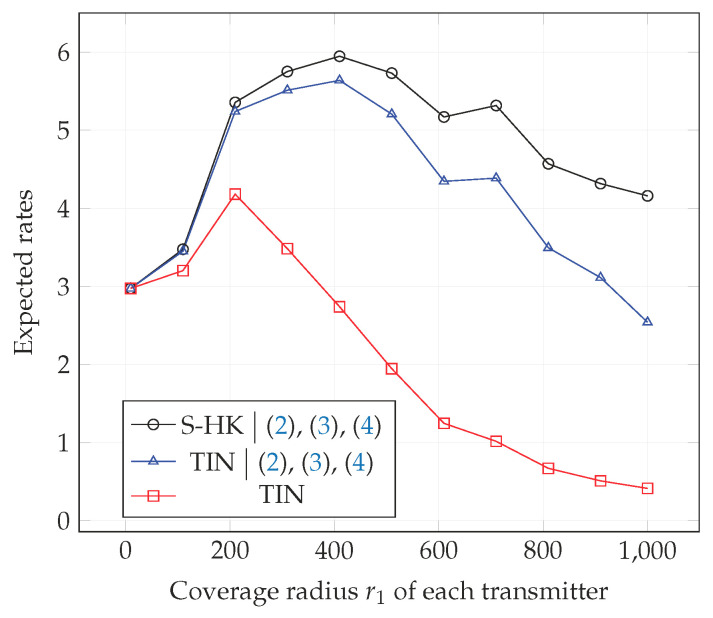
Expected rate of TIN scheme, expected rate of S-HK schemes given that the conditions (2)–(4) are satisfied and the expected rate of TIN scheme given that the conditions (2)–(4) are satisfied with topology 1, terrain type 1, K=3.

**Figure 11:**
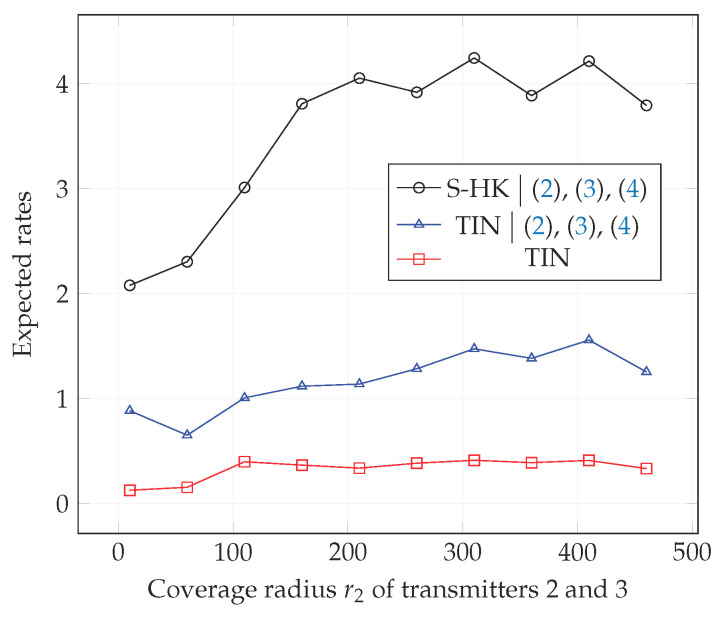
Expected rate of TIN scheme, expected rate of S-HK schemes given that the conditions (2)–(4) are satisfied and the expected rate of the TIN scheme given that the conditions (2)–(4) are satisfied with topology 2, terrain type 1, K=3.

**Figure 12:**
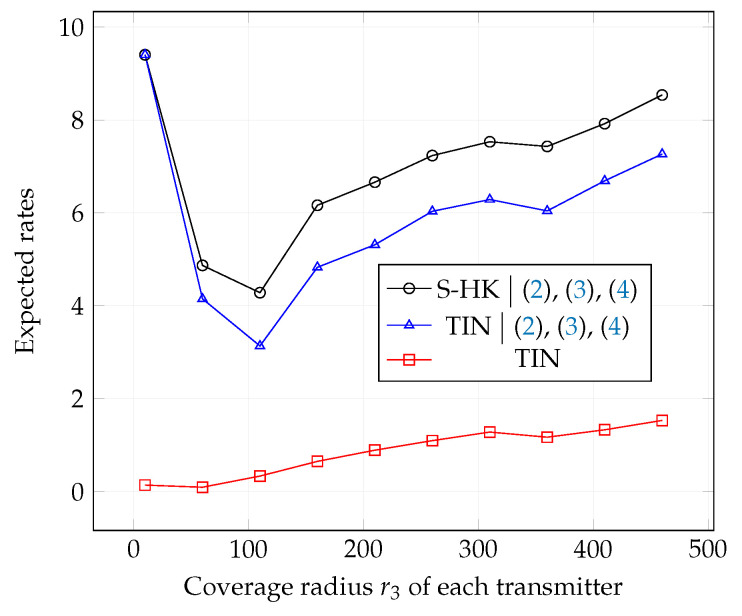
Expected rate of TIN scheme, expected rate of S-HK schemes given that the conditions (2)–(4) are satisfied and the expected rate of the TIN scheme given that the conditions (2)–(4) are satisfied with topology 3, terrain type 1, K=3.

In Figure 10, Figure 11 and Figure 12, we plot the expected rate of TIN scheme, the expected rate of S-HK schemes given that the conditions (2)–(4) are satisfied, and the expected rate of the TIN scheme given that the conditions (2)–(4) are satisfied with topologies 1, 2, 3 respectively. Note that when (2)–(4) are satisfied, S-HK schemes are optimal. Therefore, the expected rate in this case is the expected capacity. As expected, from the plots, the expected rate of TIN scheme is lower than the expected rate of S-HK schemes given that the conditions (2)–(4) are satisfied.

**Figure 13:**
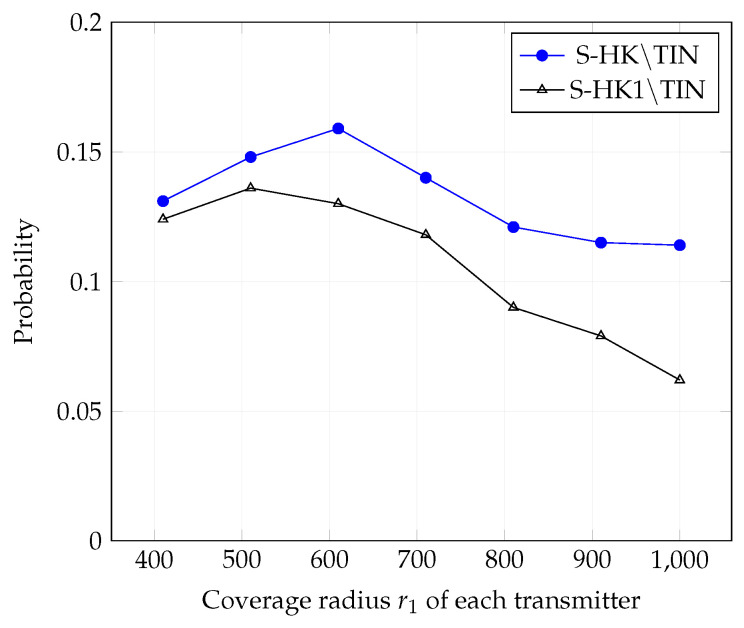
Success probability of conditions (2)–(4) for topology 1, terrain type 1, K=3.

**Figure 14:**
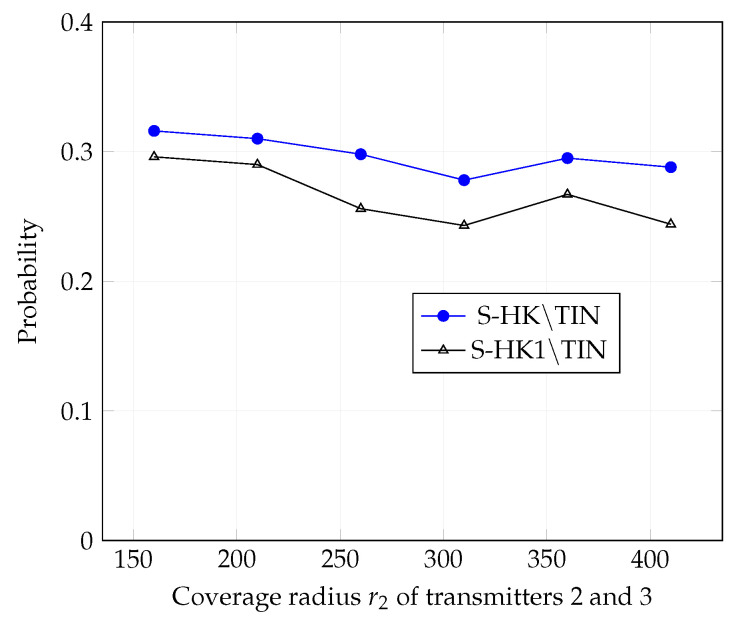
Success probability of conditions (2)–(4) for topology 2, terrain type 1, K=3.

**Figure 15:**
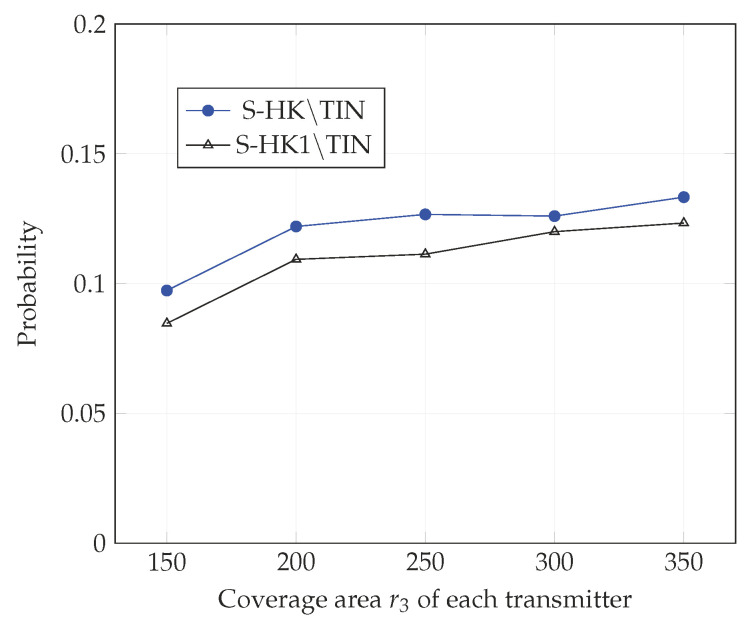
Success probability of conditions (2)–(4) for topology 3, terrain type 1, K=3.

In Figure 13, Figure 14 and Figure 15, we plot the probability that the conditions (2)–(4) are satisfied for (i) all S-HK schemes except TIN scheme, (ii) all S-HK schemes where atmost 1 strong interference signal is decoded at each receiver except TIN (denoted S-HK1∖TIN). Figure 13, Figure 14 and Figure 15 are plotted for topologies 1, 2, and 3, respectively. In topologies 2 and 3, decoding at most one strong interference at each receiver is the most important class of S-HK schemes as expected, since there is mainly one strongly interfering signal in these topologies.

**Figure 16:**
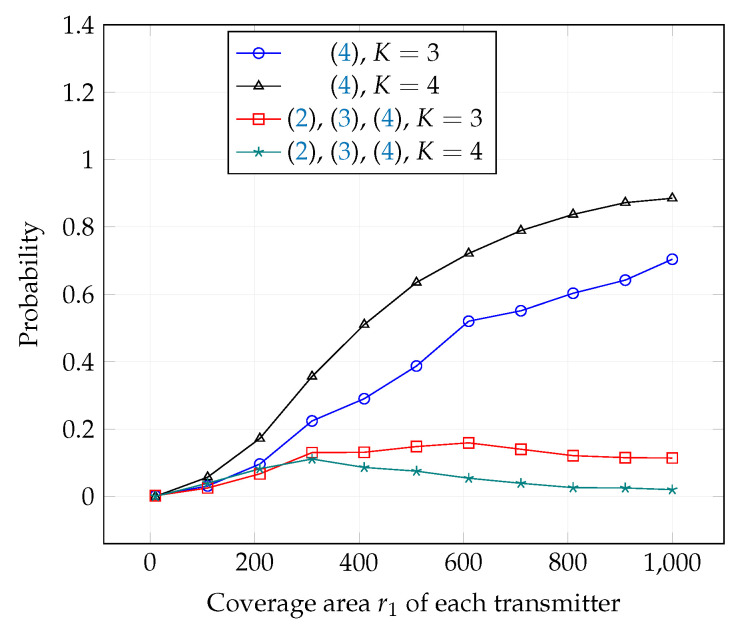
Success probability of achievability conditions (4) and success probability of conditions (2)–(4) for all S-HK schemes except TIN with topology 1, terrain type 1, K=3 and K=4.

In Figure 16, we plot the success probability of the achievability conditions (4) alone and compare them with success probability of all conditions (2)–(4) for all S-HK schemes except TIN for topology 1 with K=3 and K=4. It can be observed that the probability that achievability conditions are satisfied is much larger than the probability that all conditions are satisfied. It is worth noting that whenever the achievability conditions are satisfied, interference can be decoded and the resulting sum rate will be significantly better than the rate achieved by the TIN scheme. Therefore, even when the sum capacity conditions are not satisfied, there is significant improvement in the sum rate of S-HK schemes with interference decoding compare to the TIN scheme. As *K* increases, the probability of at least one interference signal being decodable increases as expected. Numerical results for the 2-user GIC are given in Appendix D.

## 5. Conclusions

We obtained new sum capacity results for the general *K*-user Gaussian IC. We derived two sets of channel conditions under which S-HK schemes are sum capacity optimal for the *K* user Gaussian IC. This general result also allows us to obtain all existing sum capacity results for 2-user GICs and partially connected GICs like the cascade, many-to-one and one-to-many GICs as special cases. The first sum capacity result corresponds to the case when interference is decoded and cancelled before decoding the desired signal at each receiver. The second sum capacity result corresponds to the case when one interference signal is jointly decoded with the desired message at one of the receivers. At all other receivers, interference is decoded and cancelled before the desired message.

We also studied the probability that the channel conditions required for the sum capacity result are satisfied in random wireless networks using Monte Carlo simulations. Three different random network models were considered. The numerical results showed that S-HK schemes are optimal with significant probability in the topologies that are considered. By selecting the best S-HK scheme for each channel condition, these results can be used for dynamic interference management and sum rate maximization in wireless networks. 

## Figures and Tables

**Figure A1:**
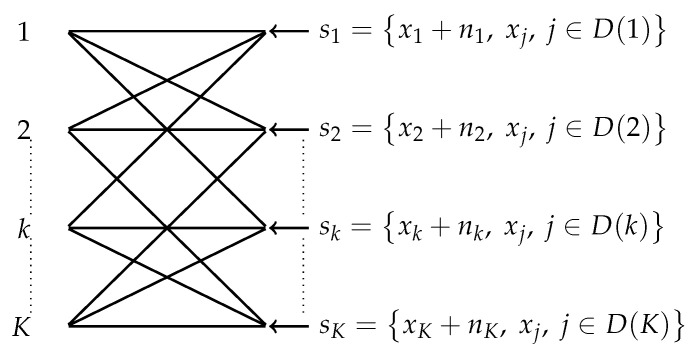
Genie aided channel for proving Theorem 1.

**Figure A2:**
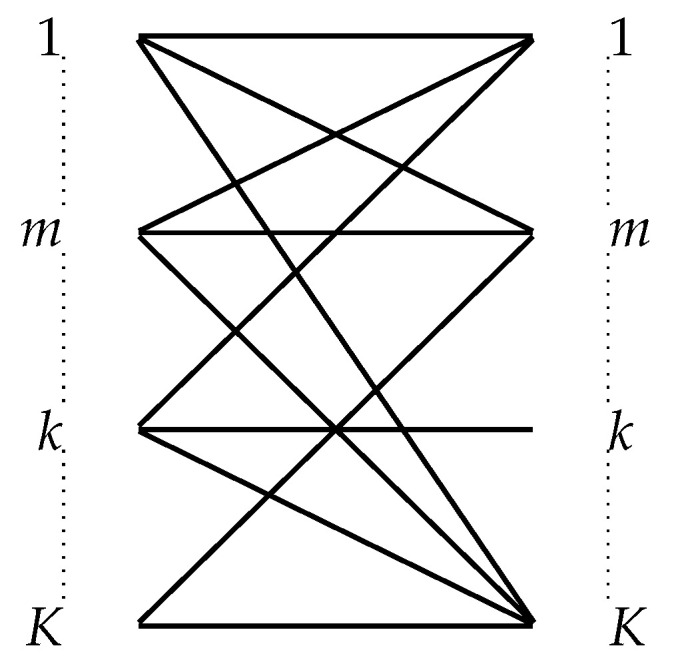
Modified channel.

**Figure A3:**
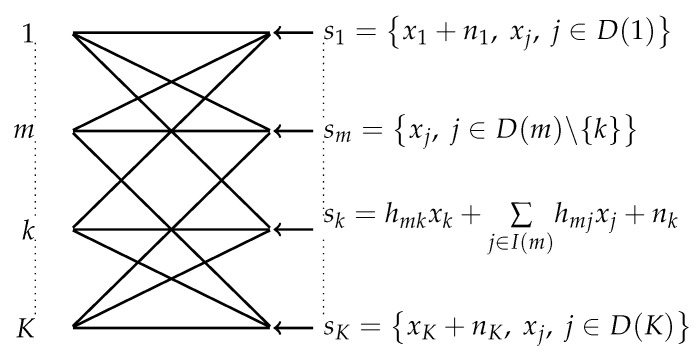
Genie aided channel for proving Theorem 3.

**Figure A4:**
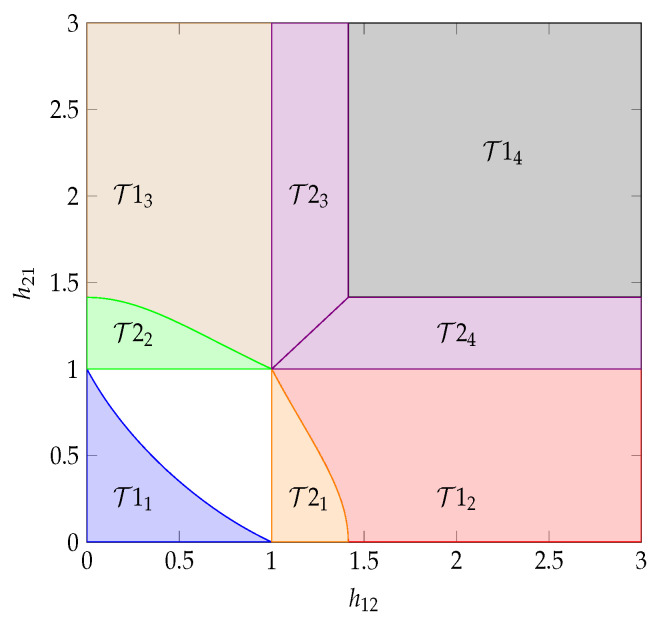
Channel conditions where sum capacity is obtained for the 2-user GIC, $P_{1}=P_{2}=1$.

**Figure A5:**
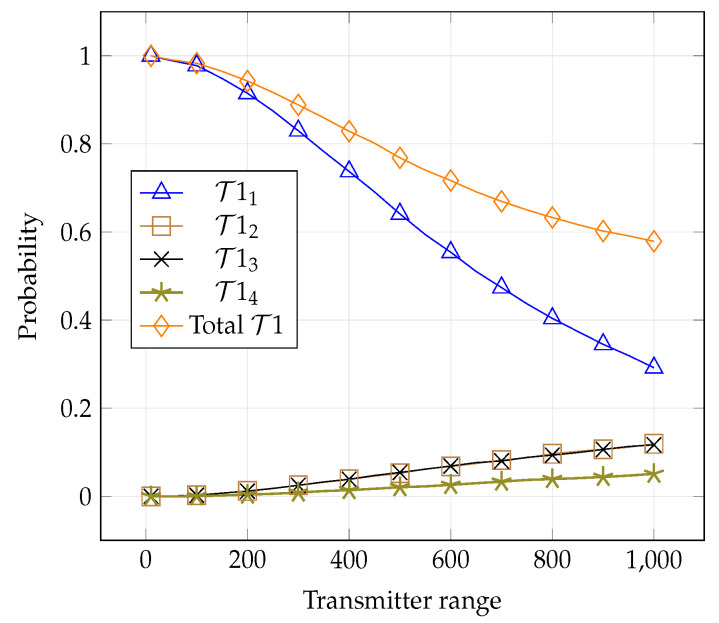
Success probability of conditions in Table A3 for Topology 1.

**Figure A6:**
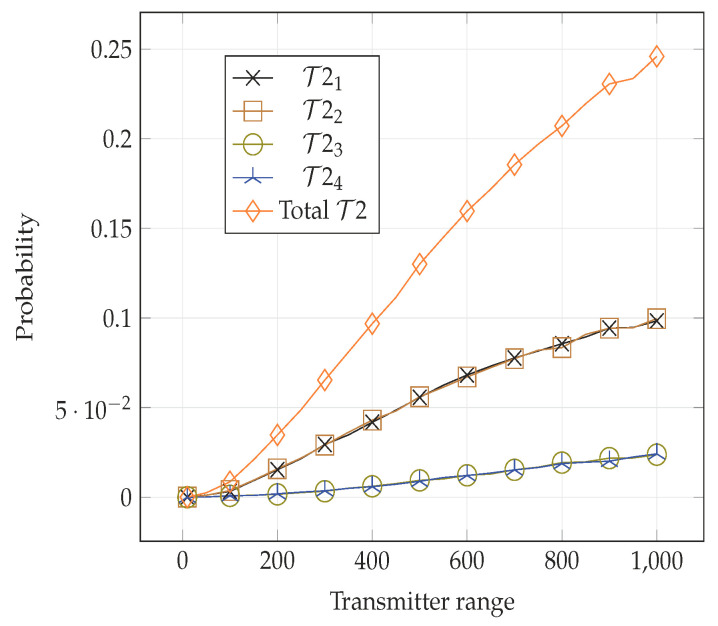
Success probability of conditions in Table A4 for Topology 1.

## Appendix A. Proof of Theorem 1

(Achievability) Suppose that each receiver *i* decodes the interference from transmitters D(i) and then decodes the information from *i*th transmitter, while treating interference from other transmitters I(i) as noise. The multiple access channel (MAC) constraints for decoding the interference at each receiver *i* are

(A1) $$\begin{array}{cc} \sum_{j\in J}R_{j} & \leq \frac{1}{2}\log\left(1+\frac{\sum_{j\in J}h_{ij}^{2}P_{j}}{1+P_{i}+Q_{i}}\right),\forall J\subseteq D\left(i\right). \end{array}$$

The sum capacity in (5) is achieved if choosing

(A2) $$R_{i}=\frac{1}{2}\log\left(1+\frac{P_{i}}{1+Q_{i}}\right),\;\forall i\in \left[K\right]$$

satisfies (A1), thereby resulting in conditions in (4).

(Converse) Consider the genie-aided channel in Figure A1, where each receiver i∈[K] is given the genie signal $s_{i}^{n}=\left\{x_{i}^{n}+n_{i}^{n},x_{j}^{n},j\in D\left(i\right)\right\}$, where $n_{i}^{n}∼N\left(0,\sigma_{i}^{2}I\right)$ and $E\left[n_{i}z_{i}\right]=\rho_{i}\sigma_{i}$, $0<\rho_{i}<1$. For the result for TIN in Reference [9], the special case of this genie-aided channel, where D(i) is empty for all *i*, was used. Now, the sum capacity can be upper bounded as

(A3) $$\begin{array}{c} nC_{sum}\leq \sum_{i=1}^{K}I\left(x_{i}^{n};y_{i}^{n},s_{i}^{n}\right)\\ =\sum_{i=1}^{K}I\left(x_{i}^{n};y_{i}^{n},x_{i}^{n}+n_{i}^{n}|x_{j}^{n},j\in D\left(i\right)\right)\\ =\sum_{i=1}^{K}\left[h\left(x_{i}^{n}+n_{i}^{n}\right)-h\left(n_{i}^{n}\right)\right]+\sum_{i=1}^{K}h\left(y_{i}^{n}|x_{i}^{n}+n_{i}^{n},x_{j}^{n},j\in D\left(i\right)\right)-\sum_{i=1}^{K}\left[h\left(\sum_{j\in I(i)}h_{ij}x_{j}^{n}+u_{i}^{n}\right)\right] \end{array}$$

where $u_{i}^{n}∼N\left(0,\left(1-\rho_{i}^{2}\right)I\right)$, ∀i∈[K]. Assuming

(A4) $$1-\rho_{i}^{2}=\phi_{i}+\sum_{j\in I(i)}h_{ij}^{2}\sigma_{j}^{2},\forall i\in \left[K\right]$$

where $\phi_{i}\geq 0$, we can write

$$\begin{array}{cc} \mathrm{cov}(u_{i}^{n}) & =\mathrm{cov}(\sqrt{\phi_{i}}n_{0}^{n}+\sum_{j\in I(i)}h_{ij}n_{j}^{n}), \end{array}$$

where $n_{0}^{n}∼N\left(0,I\right)$ and is independent of $n_{i}^{n},\forall i\in \left[K\right]$. Now, we have

(A5) $$\begin{array}{cc} \exp\left[\frac{2}{n}h\left(\sum_{j\in I(i)}h_{ij}x_{j}^{n}+u_{i}^{n}\right)\right] & =\exp\left[\frac{2}{n}h\left(\sqrt{\phi_{i}}n_{0}^{n}+\sum_{j\in I(i)}\left(h_{ij}x_{j}^{n}+h_{ij}n_{j}^{n}\right)\right)\right]\\ \; & \overset{\left(a\right)}{\geq }\exp\left[\frac{2}{n}h\left(\sqrt{\phi_{i}}n_{0}^{n}\right)\right]+\sum_{j\in I(i)}\exp\left[\frac{2}{n}h\left(h_{ij}x_{j}^{n}+h_{ij}n_{j}^{n}\right)\right]\\ \; & =2\pi e\phi_{i}+\sum_{j\in I(i)}h_{ij}^{2}\exp\left[\frac{2}{n}h\left(x_{j}^{n}+n_{j}^{n}\right)\right], \end{array}$$

where (a) follows from entropy-power inequality (EPI) [19]. Therefore, we have

$$\begin{array}{cc} \sum_{i=1}^{K}\left[h\left(x_{i}^{n}+n_{i}^{n}\right)-h\left(\sum_{j\in I(i)}h_{ij}x_{j}^{n}+u_{i}^{n}\right)\right]\; & \leq \frac{n}{2}\sum_{i=1}^{K}\left[t_{i}-\log\left(2\pi e\phi_{i}+\sum_{j\in I(i)}h_{ij}^{2}e^{t_{j}}\right)\right]\\ \; & ≜\frac{n}{2}f\left(t\right). \end{array}$$

where $t_{i}≜\frac{2}{n}h\left(x_{i}^{n}+n_{i}^{n}\right),\forall i\in \left[K\right]$ and t is the vector of all $t_{i}$’s. From the power constraints, we have $t_{i}\leq \log\left[2\pi e(P_{i}+\sigma_{i}^{2})\right],\;i\in \left[K\right].$ Under these constraints on $t_{i}$, it can be shown as in Reference [9] that f(t) is maximized at $t_{k}=\log\left[2\pi e(P_{k}+\sigma_{k}^{2})\right]$ provided $\frac{\partial f}{\partial t_{k}}$ at $t_{i}=\log\left[2\pi e(P_{i}+\sigma_{i}^{2})\right],\forall i\in \left[K\right]$ are greater than equal to 0. Thus, we have the conditions

(A6) $$\begin{array}{cc} \sum_{i:k\in I(i)}\left[\frac{h_{ik}^{2}}{1+Q_{i}-\rho_{i}^{2}}\right] & \leq \frac{1}{P_{k}+\sigma_{k}^{2}},\;\forall k\in \left[K\right]. \end{array}$$

Therefore, we now have

$$\begin{array}{cc} C_{sum} & \leq \sum_{i=1}^{K}I\left(x_{iG};y_{iG},s_{iG}\right)\\ \; & =\sum_{i=1}^{K}I\left(x_{iG};y_{iG},x_{jG},j\in D\left(i\right)\right)+\sum_{i=1}^{K}I\left(x_{iG};x_{iG}+n_{iG}|y_{iG},x_{jG},j\in D\left(i\right)\right)\\ \; & \overset{\left(b\right)}{=}\sum_{i=1}^{K}I\left(x_{iG};y_{iG},x_{jG},j\in D\left(i\right)\right)\\ \; & =\underset{i=1}{\sum^{K}}\frac{1}{2}\log\left[1+\frac{P_{i}}{1+Q_{i}}\right] \end{array}$$

where (b) is true if $I(x_{iG};x_{iG}+n_{i}|y_{iG},x_{jG},j\in D\left(i\right))=0,\forall i$. From Reference [3] (Lemma 8), $I(x_{iG};x_{iG}+n_{iG}|y_{iG},x_{jG},j\in D\left(i\right))=0$ iff

(A7) $$\rho_{i}\sigma_{i}=1+Q_{i},\forall i\in \left[K\right].$$

Also, $\phi_{i}\geq 0,\;\forall i\in \left[K\right]$ which implies

(A8) $$\begin{array}{cc} \sum_{j\in I(i)}\frac{h_{ij}^{2}{\left(1+Q_{j}\right)}^{2}}{\rho_{j}^{2}} & \leq 1-\rho_{i}^{2}, \end{array}$$

Using the conditions (A7), (A8) and (A4), we get the conditions (2) and (3) for the converse.

## Appendix B. Proof of Theorem 3

First, we consider the modified channel in Figure A2 with no interference at receiver *k*. The sum capacity of the original channel is upper bounded by the sum capacity of the modified channel.

Then, we derive a genie-aided upper bound for the modified channel in Figure A3 using the genie signals $s_{i}^{n}$ at receiver *i* for each i∈[K] as follows:

$$\begin{array}{cc} s_{i}^{n} & =\{x_{i}^{n}+n_{i}^{n},x_{j}^{n},j\in \bar{G}\left(i\right)\},\;\forall i\in \left[K\right]\setminus \left\{m,k\right\}\\ s_{m}^{n} & =\{x_{j}^{n},\;j\in \bar{G}\left(m\right)\setminus k\}\\ s_{k}^{n} & =h_{mk}x_{k}^{n}+\sum_{j\in G(m)}h_{mj}x_{j}^{n}+n_{k}^{n} \end{array}$$

where $\bar{G}\left(i\right)=\left[K\right]\setminus \left\{i,\left\{G\left(i\right)\right\}\right\}$, $n_{i}∼N\left(0,\sigma_{i}^{2}\right)$, $E\left[n_{i}z_{i}\right]=\rho_{i}\sigma_{i}$ and $0<\rho_{i}<1$, for each i∈[K]∖{m} and $\sigma_{k}=1$. This choice of genie can be shown to be useful and smart under conditions (7)–(10) to obtain the upper bound in the theorem statement. This proof generalizes the proof for the many-to-one GIC in Reference [13] (Theorem 4) to the general *K* user GIC. Assume

(A9) $$\begin{array}{cc} 1-\rho_{i}^{2} & =\phi_{i}+\sum_{j\in G(i)}h_{ij}^{2}\sigma_{j}^{2},i\in \left[K\right]\setminus \left\{m,k\right\}, \end{array}$$

(A10) $$\begin{array}{cc} 1-\rho_{k}^{2} & =\phi_{k}+\sum_{j\in G(m)}h_{mj}^{2}\sigma_{j}^{2}, \end{array}$$

where $\phi_{i}\geq 0$ and assume $h_{ij}^{2}\leq 1,\;\;\forall i\in G\left(i\right),\;i\in \left[K\right]$. Now, we have

$$\begin{array}{cc} nC_{sum}\leq \sum_{i=1}^{K}I\left(x_{i}^{n};y_{i}^{n},s_{i}^{n}\right)\\ \; & =I\left(x_{m}^{n};y_{m}^{n}|s_{m}^{n}\right)+I\left(x_{k}^{n};y_{k}^{n},s_{k}^{n}\right)+\sum_{\underset{i\neq \{m,k\}}{i=1}}^{K}I\left(x_{i}^{n};y_{i}^{n},s_{i}^{n}\right)\\ \; & =h\left(y_{m}^{n}|s_{m}^{n}\right)-h\left(y_{m}^{n}|s_{m}^{n},x_{m}^{n}\right)+h\left(s_{k}^{n}\right)+h\left(y_{k}^{n}|s_{k}^{n}\right)-h\left(z_{k}^{n}\right)-h\left(s_{k}^{n}|y_{k}^{n},x_{k}^{n}\right)\\ \; & \;\;+\sum_{\underset{i\neq \{m,k\}}{i=1}}^{K}I\left(x_{i}^{n};y_{i}^{n},s_{i}^{n}\right)\\ \; & \overset{\left(a\right)}{=}h\left(y_{m}^{n}|s_{m}^{n}\right)+h\left(y_{k}^{n}|s_{k}^{n}\right)-h\left(z_{k}^{n}\right)-h\left(\sum_{j\in G(m)}h_{mj}x_{j}^{n}+u_{k}^{n}\right)\\ \; & +\sum_{\underset{i\neq \{m,k\}}{i=1}}^{K}\left[h\left(x_{i}^{n}+n_{i}^{n}\right)\right]+\sum_{\underset{i\neq \{m,k\}}{i=1}}^{K}\left[h\left(y_{i}^{n}|s_{i}^{n}\right)-h\left(n_{i}^{n}\right)-h\left(\sum_{j\in G(i)}h_{ij}x_{j}^{n}+u_{i}^{n}\right)\right] \end{array}$$

where (a) follows because $h\left(s_{k}^{n}\right)=h\left(y_{m}^{n}|s_{m}^{n},x_{m}^{n}\right)$, $u_{i}^{n}∼N\left(0,\left(1-\rho_{i}^{2}\right)I\right)$, ∀i∈[K]∖{m}. Also note that from (A9) and (A10), we have

$$\begin{array}{cc} \mathrm{cov}(u_{i}^{n}) & =\mathrm{cov}\left(\sqrt{\phi_{i}}n_{0}^{n}+\sum_{j\in G(i)}h_{ij}n_{j}^{n}\right)\forall i\in \left[K\right]\setminus \left\{m,k\right\},\\ \mathrm{cov}(u_{k}^{n}) & =\mathrm{cov}(\sqrt{\phi_{k}}n_{0}^{n}+\sum_{j\in G(m)}h_{mj}n_{j}^{n}), \end{array}$$

where $n_{0}^{n}∼N\left(0,I\right)$ and $n_{0}^{n}$ is independent of $n_{i}^{n},\;i\in \left[K\right]\setminus \left\{m\right\}$. From EPI, we have

$$\begin{array}{cc} \exp\left[\frac{2}{n}h\left(\sum_{j\in G(i)}h_{ij}x_{j}^{n}+u_{i}^{n}\right)\right] & =\exp\left[\frac{2}{n}h\left(\sqrt{\phi_{i}}n_{0}^{n}+\sum_{j\in G(i)}\left(h_{ij}x_{j}^{n}+h_{ij}n_{j}^{n}\right)\right)\right]\\ \; & \geq \exp\left[\frac{2}{n}h\left(\sqrt{\phi_{i}}n_{0}^{n}\right)\right]+\sum_{j\in G(i)}\exp\left[\frac{2}{n}h\left(h_{ij}x_{j}^{n}+h_{ij}n_{j}^{n}\right)\right] \end{array}$$

(A11) $$\begin{array}{cc} \; & =2\pi e\phi_{i}+\sum_{j\in G(i)}\exp\left[\frac{2}{n}h\left(x_{j}^{n}+n_{j}^{n}\right)\right]\exp\left[\frac{2}{n}\log\left(h_{ij}^{n}\right)\right] \end{array}$$

(A12) $$\begin{array}{cc} \; & =2\pi e\phi_{i}+\sum_{j\in G(i)}h_{ij}^{2}\exp\left[\frac{2}{n}h\left(x_{j}^{n}+n_{j}^{n}\right)\right] \end{array}$$

Considering the terms that are not directly maximized by Gaussian inputs, we get

$$\begin{array}{cc} \; & \sum_{\underset{i\neq m,k}{i=1}}^{K}\left[h\left(x_{i}^{n}+n_{i}^{n}\right)-h\left(\sum_{j\in G(i)}h_{ij}x_{j}^{n}+u_{i}^{n}\right)\right]-h\left(\sum_{j\in G(m)}h_{mj}x_{j}^{n}+u_{k}^{n}\right)\\ \; & \overset{\left(b\right)}{\leq }\frac{n}{2}\sum_{\underset{i\neq m,k}{i=1}}^{K}\left[t_{i}-\log\left(2\pi e\phi_{i}+\sum_{j\in G(i)}h_{ij}^{2}e^{t_{j}}\right)\right]-\frac{n}{2}\log\left(2\pi e\phi_{k}+\sum_{j\in G(m)}h_{mj}^{2}e^{t_{j}}\right)\\ \; & ≜\frac{n}{2}f\left(t\right), \end{array}$$

where (b) follows from (A12) and $t_{i}≜\frac{2}{n}h\left(x_{i}^{n}+n_{i}^{n}\right),\;i\in \left[K\right]$. From the power constraints, we have

$$t_{i}\leq \log\left[2\pi e(P_{i}+\sigma_{i}^{2})\right],\;i=1,\cdots ,K.$$

Since m,k∉G(i), ∀i∈[K]∖{k}, terms $t_{m}$, $t_{k}$ do not appear in f(t) and we consider the optimization problem

$$\begin{array}{cc} \max\; & f(t)\\ s.t\; & t_{i}\leq \log\left[2\pi e(P_{i}+\sigma_{i}^{2})\right],\\ \; & \forall i\in [K]\setminus \{m,k\}. \end{array}$$

The Lagrangian for the above optimization problem is

$$L=f\left(t\right)-\sum_{i\neq m,k}\mu_{i}\left[t_{i}-\log\left[2\pi e(P_{i}+\sigma_{i}^{2})\right]\right],$$

where $\mu_{i}\geq 0,\forall i\in \left[K\right]$. At optimal $t_{r},\frac{\partial L}{\partial t_{r}}=0$. We want optimal $t_{r}=\frac{1}{2}\log\left[2\pi e(P_{r}+\sigma_{r}^{2})\right]$ for Gaussian inputs to be optimal for the genie-aided channel. From KKT conditions, $\frac{\partial f}{\partial t_{r}}\geq 0$ at $t_{r}=\log\left[2\pi e(P_{r}+\sigma_{r}^{2})\right],\forall r\in \left[K\right]\setminus \left\{m,k\right\}$ (since $\mu_{r}\geq 0$). Thus, we have

$$\frac{1}{P_{r}+\sigma_{r}^{2}}\geq \sum_{\underset{i\neq \{m,k\}}{i:r\in G(i)}}\left[\frac{h_{ir}^{2}}{1+Q_{i}-\rho_{i}^{2}}\right]+\delta_{r}\left[\frac{h_{mr}^{2}}{1+Q_{m}-\rho_{k}^{2}}\right],\forall \;r\in \left[K\right]\setminus \left\{m,k\right\},$$

where $Q_{i},\delta_{r}$ are defined as in the theorem statement. Therefore, we now have

$$\begin{array}{cc} C_{sum} & \leq \sum_{i=1}^{K}I\left(x_{iG};y_{iG},s_{iG}\right)\\ \; & =I\left(x_{mG};y_{mG}|s_{mG}\right)+I\left(x_{kG};y_{kG},s_{kG}\right)+\sum_{\underset{i\neq \{m,k\}}{i=1}}^{K}I\left(x_{iG};y_{iG},s_{iG}\right)\\ \; & \overset{\left(c\right)}{=}I\left(x_{mG};y_{mG}|s_{mG}\right)+I\left(x_{kG};s_{kG}\right)+\sum_{\underset{i\neq \{m,k\}}{i=1}}^{K}I\left(x_{iG};y_{iG}|x_{jG},j\in \bar{G}\left(i\right)\right)\\ \; & \overset{\left(d\right)}{=}I\left(x_{mG},x_{kG};y_{mG}|s_{mG}\right)+\sum_{\underset{i\neq \{m,k\}}{i=1}}^{K}I\left(x_{iG};y_{iG}|x_{jG},j\in \bar{G}\left(i\right)\right), \end{array}$$

(c) is valid when $I(x_{iG};x_{iG}+n_{i}|y_{iG},x_{jG},j\in \bar{G}\left(i\right))=0$, ∀i∈[K]∖{m,k} and $I(x_{kG};y_{kG}|s_{kG})=0$. From Reference [3] (Lemma 8), $I\left(x_{iG};x_{iG}+n_{i}|y_{iG},x_{jG},j\in \bar{G}\left(i\right)\right)=I\left(x_{iG};x_{iG}+n_{i}|\left(x_{iG}+\sum_{j\in I(i)}h_{ij}x_{jG}+z_{i}\right)\right)=0$, ∀i∈[K]∖{m,k} and $I\left(x_{kG};y_{kG}|s_{kG}\right)=I\left(x_{kG};x_{kG}+z_{k}|\frac{x_{kG}+\sum_{j\in I(m)}h_{mj}x_{jG}+n_{k}}{h_{mk}}\right)=0$ iff

(A13) $$\begin{array}{cc} \rho_{i}\sigma_{i} & =1+Q_{i},\forall i\in \left[K\right]\setminus \left\{m,k\right\}, \end{array}$$

(A14) $$\begin{array}{cc} \rho_{k}h_{mk} & =1+Q_{m}. \end{array}$$

(d) is valid since genie $s_{k}$ is chosen such that $h\left(s_{k}\right)=h\left(y_{m}|s_{m},x_{m}\right)$ which implies $I\left(x_{kG};s_{kG}\right)=I\left(x_{kG};y_{mG}|s_{mG},x_{mG}\right)$.

Using $\phi_{i}\geq 0$ and (A13) and (A14), we get conditions (7)–(10) for the upper bound on $C_{sum}$ to be valid.

## Appendix C. Examples

In this section, we will give some examples of finding the two sets of channel conditions under which sum capacity is achieved by a S-HK scheme using Theorems 1–3.

**Example** **A1.**
*In this example, using Theorem 1, we will find the first set of channel conditions under which sum capacity is achieved by a S-HK scheme. Consider a 3-user GIC with S-HK scheme given by I(1)={2}, I(2)={3}, I(3)={}. Inequalities (2) and (3) gives the same set of conditions given by*

(A15) $$\begin{array}{cc} h_{12}^{2}\left(1+h_{23}^{2}P_{3}\right) & \leq \rho_{2}^{2}\left(1-\rho_{1}^{2}\right) \end{array}$$

(A16) $$\begin{array}{cc} h_{23}^{2} & \leq (1-\rho_{2}^{2}) \end{array}$$

*for some $\rho_{2},\rho_{1}\in \left(0,1\right)$. In inequality (4), for i=1, J={3}; for i=2, J={1}; and for i=3, J can be {1}, {2} and {1,2}. Therefore, (4) gives the set of conditions*

(A17) $$\begin{array}{cc} 1+P_{1}+h_{12}^{2}P_{2} & \leq h_{13}^{2} \end{array}$$

(A18) $$\begin{array}{cc} 1+P_{2}+h_{23}^{2}P_{3} & \leq h_{21}^{2}\left(1+h_{12}^{2}P_{2}\right) \end{array}$$

(A19) $$\begin{array}{cc} \left(1+P_{3}\right) & \leq h_{31}^{2}\left(1+h_{12}^{2}P_{2}\right) \end{array}$$

(A20) $$\begin{array}{cc} \left(1+P_{3}\right) & \leq h_{32}^{2}\left(1+h_{23}^{2}P_{3}\right) \end{array}$$

(A21) $$\begin{array}{cc} \left(1+\frac{P_{1}}{1+Q_{1}}\right)\left(1+\frac{P_{2}}{1+Q_{2}}\right) & \leq \left(1+\frac{h_{31}^{2}P_{1}+h_{32}^{2}P_{2}}{1+P_{3}}\right) \end{array}$$

*where $Q_{1}=h_{12}^{2}P_{2}$, $Q_{2}=h_{23}^{2}P_{3}$, $Q_{3}=0$. Under conditions (A15)–(A21), sum capacity is achieved by S-HK scheme with I(1)={2}, I(2)={3}, I(3)={} and the sum capacity is given by*

$$C_{sum}=\sum_{i=1}^{3}\frac{1}{2}\log\left[1+\frac{P_{i}}{1+Q_{i}}\right].$$

**Example** **A2.**
*In this example, we will find achievable sum rates in (6) for a S-HK scheme. Consider a 3-user GIC with S-HK scheme given by I(1)={}, I(2)={1,3}, I(3)={2} which implies D(1)={2,3}, D(2)={}, D(3)={1}. From Theorem 2, l can be 1,2,3 and $J_{1}\subseteq \left\{1,2,3\right\}$, i.e., $J_{1}$ can be {}, {1}, {2}, {3}, {1,2},{1,3},{2,3}, {1,2,3}. $J_{2}\subseteq \left\{2\right\}$, $J_{3}\subseteq \left\{1,3\right\}$. For l=1,2,3, the possible sets of $J_{1}$, $J_{2}$, $J_{3}$ such that $\underset{i\in \left[K\right]}{⋃}J_{i}=S_{l}$ are given in Table A1.*

**Table A1:** *Set of $J_{i}$ such that $\underset{i\in \left[K\right]}{⋃}J_{i}=S_{l}$ for S-HK with I(1)={}, I(2)={1,3}, I(3)={2}*.

*l*	$J_{1}$	$J_{2}$	$J_{3}$
l=1	{}	{2}	{1,3}
	{1}	{2}	{3}
	{2}	{}	{1,3}
	{3}	{2}	{1}
	{1,2}	{}	{3}
	{1,3}	{2}	{}
	{2,3}	{}	{1}
	{1,2,3}	{}	{}
l=2	{1,2,3}	{2}	{1,3}
*Achievable sum rates are given by*

$$\begin{array}{cc} S & \leq \frac{1}{2}\log\left[1+\frac{P_{2}}{1+Q_{2}}\right]+\frac{1}{2}\log\left[1+\frac{h_{31}^{2}P_{1}+P_{3}}{1+Q_{3}}\right]\\ S & \leq \frac{1}{2}\log\left(1+P_{1}\right)+\frac{1}{2}\log\left[1+\frac{P_{2}}{1+Q_{2}}\right]+\frac{1}{2}\log\left[1+\frac{P_{3}}{1+Q_{3}}\right]\\ S & \leq \frac{1}{2}\log\left(1+h_{12}^{2}P_{2}\right)+\frac{1}{2}\log\left[1+\frac{h_{31}^{2}P_{1}+P_{3}}{1+Q_{3}}\right]\\ S & \leq \frac{1}{2}\log\left(1+h_{13}^{2}P_{3}\right)+\frac{1}{2}\log\left[1+\frac{P_{2}}{1+Q_{2}}\right]+\frac{1}{2}\log\left[1+\frac{h_{31}^{2}P_{1}}{1+Q_{3}}\right]\\ S & \leq \frac{1}{2}\log\left(1+P_{1}+h_{12}^{2}P_{2}\right)+\frac{1}{2}\log\left[1+\frac{P_{3}}{1+Q_{3}}\right]\\ S & \leq \frac{1}{2}\log\left(1+P_{1}+h_{13}^{2}P_{3}\right)+\frac{1}{2}\log\left[1+\frac{P_{2}}{1+Q_{2}}\right]\\ S & \leq \frac{1}{2}\log\left(1+h_{12}^{2}P_{2}+h_{13}^{2}P_{3}\right)+\frac{1}{2}\log\left[1+\frac{h_{31}^{2}P_{1}}{1+Q_{3}}\right]\\ S & \leq \frac{1}{2}\log\left(1+P_{1}+h_{12}^{2}P_{2}+h_{13}^{2}P_{3}\right)\\ 2S & \leq \frac{1}{2}\log\left(1+P_{1}+h_{12}^{2}P_{2}+h_{13}^{2}P_{3}\right)+\frac{1}{2}\log\left[1+\frac{P_{2}}{1+Q_{2}}\right]+\frac{1}{2}\log\left[1+\frac{h_{31}^{2}P_{1}+P_{3}}{1+Q_{3}}\right]. \end{array}$$

*where $Q_{2}=h_{21}^{2}P_{1}+h_{23}^{2}P_{3}$, $Q_{3}=h_{23}^{2}P_{2}$. Depending on the channel and power constraints one of the above inequalities will be dominant.*

**Example** **A3.**
*In this example, using Theorems 2 and 3 we will find the second set of channel conditions under which sum capacity is achieved by a S-HK scheme. Consider a 3-user GIC with S-HK scheme given by I(1)={3}, I(2)={1,3}, I(3)={}. Let m=1, k=2. Here m, k∉I(i), i∈{1,3}. First we will find the converse conditions or the conditions under which sum rate*

(A22) $$S\leq \frac{1}{2}\log\left[1+\frac{\left(P_{1}+h_{12}^{2}P_{2}\right)}{1+Q_{1}}\right]+\frac{1}{2}\log\left(1+P_{3}\right)$$

*is an upper bound. For I(1)={3}, I(2)={1,3}, I(3)={}, inequalities (7) and (10) gives the same set of conditions*

(A23) $$\begin{array}{cc} h_{13}^{2} & \leq \rho_{3}^{2}\left(1-\rho_{2}^{2}\right). \end{array}$$

*(9) does not give any condition. (8) implies*

(A24) $$\begin{array}{cc} \rho_{2}h_{12} & =1+h_{13}^{2}P_{3} \end{array}$$

*Combining (A23) and (A24), sum rate in (A22) is an upper bound for all the channels satisfying*

(A25) $$h_{13}^{2}+{\left(\frac{1+h_{13}^{2}P_{3}}{h_{12}}\right)}^{2}\leq 1$$

*For achievablity conditions, first we will find all achievable sum rates of the S-HK scheme using Theorem 2. Observe that $J_{1}\subseteq \left\{1,2\right\}$, $J_{2}\subseteq \left\{2\right\}$, $J_{3}\subseteq \left\{1,2,3\right\}$. For l=1,2,3, the possible sets of $J_{1}$, $J_{2}$, $J_{3}$ such that $\underset{i\in \left[K\right]}{⋃}J_{i}=S_{l}$ are given in Table A2.*

**Table A2:** *Set of $J_{i}$ such that $\underset{i\in \left[K\right]}{⋃}J_{i}=S_{l}$ for S-HK with I(1)={3}, I(2)={1,3}, I(3)={}*.

*l*	$J_{1}$	$J_{2}$	$J_{3}$
l=1	{}	{}	{1,2,3}
	{}	{2}	{1,3}
	{1}	{}	{2,3}
	{1}	{2}	{3}
	{2}	{}	{1,3}
	{1,2}	{}	{3}
*Achievable sum rates for S-HK with I(1)={3}, I(2)={1,3}, I(3)={} are given by*

(A26) $$\begin{array}{cc} S & \leq \frac{1}{2}\log\left[1+P_{3}+h_{32}^{2}P_{2}+h_{31}^{2}P_{1}\right] \end{array}$$

(A27) $$\begin{array}{cc} S & \leq \frac{1}{2}\log\left[1+\frac{P_{2}}{1+Q_{2}}\right]+\frac{1}{2}\log\left[1+P_{3}+h_{31}^{2}P_{1}\right] \end{array}$$

(A28) $$\begin{array}{cc} S & \leq \frac{1}{2}\log\left[1+\frac{P_{1}}{1+Q_{1}}\right]+\frac{1}{2}\log\left[1+P_{3}+h_{32}^{2}P_{2}\right] \end{array}$$

(A29) $$\begin{array}{cc} S & \leq \frac{1}{2}\log\left[1+\frac{P_{1}}{1+Q_{1}}\right]+\frac{1}{2}\log\left[1+\frac{P_{2}}{1+Q_{2}}\right]+\frac{1}{2}\log\left(1+P_{3}\right) \end{array}$$

(A30) $$\begin{array}{cc} S & \leq \frac{1}{2}\log\left[1+\frac{h_{12}^{2}P_{2}}{1+Q_{1}}\right]+\frac{1}{2}\log\left[1+P_{3}+h_{31}^{2}P_{1}\right] \end{array}$$

(A31) $$\begin{array}{cc} S & \leq \frac{1}{2}\log\left[1+\frac{P_{1}+h_{12}^{2}P_{2}}{1+Q_{1}}\right]+\frac{1}{2}\log\left(1+P_{3}\right) \end{array}$$

*where $Q_{1}=h_{13}^{2}P_{3}$, $Q_{2}=h_{21}^{2}P_{1}+h_{23}^{2}P_{3}$. We want (A31) to be dominant among the inequalities (A26)–(A31) which gives the conditions*

(A32) $$\begin{array}{cc} \left(1+P_{3}\right)\left(P_{1}+h_{12}^{2}P_{2}\right) & \leq \left(h_{32}^{2}P_{2}+h_{31}^{2}P_{1}\right)\left(1+Q_{1}\right) \end{array}$$

(A33) $$\begin{array}{cc} \left(1+P_{3}\right)\left(\frac{P_{1}+h_{12}^{2}P_{2}}{1+Q_{1}}\right) & \leq \frac{\left(1+P_{3}+h_{31}^{2}P_{1}\right)P_{2}}{1+Q_{2}}+h_{31}^{2}P_{1} \end{array}$$

(A34) $$\begin{array}{cc} h_{12}^{2}\left(1+P_{3}\right) & \leq h_{32}^{2}\left(1+P_{1}+Q_{1}\right) \end{array}$$

(A35) $$\begin{array}{cc} h_{12}^{2}\left(1+Q_{2}\right) & \leq (1+P_{1}+Q_{1}) \end{array}$$

(A36) $$\begin{array}{cc} \left(1+P_{3}\right) & \leq \left(1+Q_{1}+h_{12}^{2}P_{2}\right)h_{31}^{2} \end{array}$$

*Therefore, under conditions (A32)–(A36) and (A25), sum capacity is achievable by S-HK scheme with I(1)={3}, I(2)={1,3}, I(3)={} and the sum capacity is given by (A22).*

## Appendix D. 2-User GIC Results

For a 2 user GIC, there are only 4 possible S-HK schemes. Optimality conditions and sum capacity of S-HK schemes using Theorem 1 are given in Table A3.

**Table A3:** Optimality conditions and sum capacity of S-HK schemes for a 2 user GIC using Theorem 1.

S-HK Scheme	Optimality Conditions	Sum Capacity	Matches
I(1)={2}	$|h_{12}\left(1+h_{21}^{2}P_{1}\right)|+$	$\frac{1}{2}\log\left(1+\frac{P_{1}}{1+h_{12}^{2}P_{1}}\right)$	
I(2)={1}	$|h_{21}\left(1+h_{12}^{2}P_{2}\right)|\leq 1$	$+\frac{1}{2}\log\left(1+\frac{P_{2}}{1+h_{21}^{2}P_{1}}\right)$	[3,4,6]
I(1)={}	$h_{21}^{2}\leq 1$,	$\frac{1}{2}\log\left(1+P_{1}\right)+$	Thm. 10 in [6]
I(2)={1}	$h_{12}^{2}\geq \frac{1+P_{1}}{1+h_{21}^{2}P_{1}}$	$\frac{1}{2}\log\left(1+\frac{P_{2}}{1+h_{21}^{2}P_{1}}\right)$	
I(1)={2}	$h_{12}^{2}\leq 1$,	$\frac{1}{2}\log\left(1+P_{2}\right)+$	Thm. 10 in [6]
I(2)={}	$h_{21}^{2}\geq \frac{1+P_{2}}{1+h_{12}^{2}P_{2}}$	$\frac{1}{2}\log\left(1+\frac{P_{1}}{1+h_{12}^{2}P_{2}}\right)$	
I(1)={}	$h_{12}^{2}\geq 1+P_{1}$,	$\frac{1}{2}\log\left(1+P_{1}\right)+$	[1]
I(2)={}	$h_{21}^{2}\geq 1+P_{2}$	$\frac{1}{2}\log\left(1+P_{2}\right)$	

Optimality conditions and sum capacity of S-HK schemes using Theorems 2 and 3 are given in Table A4. These conditions are plotted in Figure A4.

In Figure A4, $T1_{i}$ denotes the region given by scheme *i* (corresponds to *i*th row in the Table) in Table A3 and $T2_{i}$ denotes the region given by scheme *i* in Table A4. In Figure A5 and Figure A6, for Topology 1, we plot the success probability of conditions in Table A3 and Table A4 respectively. For topology 1, as transmitter range increases, the probability that decoding interference is optimal increases and the probability that treating interference as noise is optimal reduces. Therefore, as expected, probability that $T1_{1}$ is optimal decreases with increasing range and the probability that the other schemes are optimal increases.

**Table A4:** Optimality conditions and sum capacity of S-HK schemes for a 2 user GIC using Theorems 2 and 3.

S-HK Scheme	Optimality Conditions	Sum Capacity	Matches
I(1)={}	$h_{21}\leq 1,h_{12}\geq 1$,	$\frac{1}{2}\log\left(1+P_{1}+h_{12}^{2}P_{2}\right)$	Thm. 10 in Reference [6]
I(2)={1}	$h_{12}^{2}\left(1+h_{21}^{2}P_{1}\right)\leq 1+P_{1}$		
I(1)={2}	$h_{12}\leq 1,h_{21}\geq 1$,	$\frac{1}{2}\log\left(1+P_{2}+h_{21}^{2}P_{1}\right)$	Thm. 10 in Reference [6]
I(2)={}	$h_{21}^{2}\left(1+h_{12}^{2}P_{2}\right)\leq 1+P_{2}$		
I(1)={}	$1\leq h_{12}^{2}\leq 1+P_{1}$,	$\frac{1}{2}\log\left(1+P_{1}+h_{12}^{2}P_{2}\right)$	
I(2)={}	$P_{1}+h_{12}^{2}P_{2}\leq P_{2}+h_{21}^{2}P_{1}$		
			[2]
	$1\leq h_{21}^{2}\leq 1+P_{2}$,	$\frac{1}{2}\log\left(1+P_{2}+h_{21}^{2}P_{1}\right)$	
	$P_{2}+h_{21}^{2}P_{1}\leq P_{1}+h_{12}^{2}P_{2}$		

## Appendix E. Partially Connected GIC Results

We specialize our sum capacity results to cyclic, cascade and many-to-one GICs, which are special cases of GIC. For cyclic channels, we specialize the two channel conditions for general S-HK schemes to get two new sum capacity results. For 3 user cascade channels, sum capacity results were derived in Reference [11] but we derive sum capacity results for a general *K* user cascade channels. For the many-to-one and one-to-many GICs, we can recover the results derived in Reference [13].

### Appendix E.1. Cyclic GIC

We use the following channel model for cyclic GIC

$$y_{k}=x_{k}+h_{k+1}x_{k+1}+z_{k},\forall k\in \left[K\right],$$

where the indices are modulo *K*.

**Result** **A1.** *For a cyclic GIC, satisfying the following conditions for some sets*$I_{1},D_{1}\subseteq \left[K\right]$*and*$I_{1}\cup D_{1}=\left[K\right]$

(A37) $$\begin{array}{cc} \frac{h_{i+1}^{2}{\left(1+Q_{i+1}\right)}^{2}}{\rho_{i+1}^{2}} & \leq 1-\rho_{i}^{2},\;\forall i\in I_{1} \end{array}$$

(A38) $$\begin{array}{cc} h_{i+1}^{2}\left(1+Q_{i+1}\right) & \geq 1+P_{i},\forall i\in D_{1} \end{array}$$

*where*

$$Q_{i}=\left\{\begin{array}{cc} h_{i+1}^{2}P_{i+1} & :i\in I_{1}\\ 0 & :else \end{array}\right.$$

*the sum capacity is given by*

(A39) $$C_{sum}=\underset{i=1}{\sum^{K}}\frac{1}{2}\log\left[1+\frac{P_{i}}{1+Q_{i}}\right]$$

*and the sum capacity is achieved by S-HK scheme defined by*$I\left(i\right)=\phi ,\;\forall i\in D_{1}$*and*$I\left(i\right)=\left\{i+1\right\},\;\forall i\in I_{1}$.

**Proof.** Use Theorem 1. □

**Corollary** **A1.**
*For the cyclic channel, if we treat interference as noise at receivers $i\in I_{1}$ and decode interference at receivers $i\in D_{1}=\left[K\right]\setminus I_{1}$, then the achievable sum rates given by*

$$\begin{array}{cc} S & \leq \frac{1}{2}\sum_{j\in J_{1}}\log\left(1+P_{j}+h_{j+1}^{2}P_{j+1}\right)+\frac{1}{2}\sum_{j\in J_{2}}\log\left(1+c_{j}P_{j}\right)\\ \; & \;\;\forall J_{1}\subseteq D_{1},\;such\;that\;if\;\;i\in J_{1},\;then,\;i+1\notin J_{1} \end{array}$$

(A40) $$\begin{array}{cc} \; & \;\;J_{2}=\left[K\right]\setminus \left\{i,i+1:i\in J_{1}\right\} \end{array}$$

(A41) $$\begin{array}{cc} 2S & \leq \frac{1}{2}\sum_{j=1}^{K}\log\left(1+P_{j}+h_{j+1}^{2}P_{j+1}\right),if\;D_{1}=\left[K\right] \end{array}$$

*where, for all i=1,2,⋯,K*

$$c_{i}=\left\{\begin{array}{cc} \min\left\{h_{i}^{2},\frac{1}{1+h_{i+1}^{2}P_{i+1}}\right\} & :i-1\in D_{1},\;i\in I_{1}\\ \min\{h_{i}^{2},1\} & :i-1\in D_{1},\;i\in D_{1}\\ \frac{1}{1+h_{i+1}^{2}P_{i+1}} & :i-1\in I_{1},\;i\in I_{1}\\ 1 & :i-1\in I_{1},\;i\in D_{1} \end{array}\right.$$

*are achievable.*

**Proof.** Use Theorem 2. □

**Result** **A2.** *For the cyclic GIC, let*$I_{1},D_{1}\subseteq \left[K\right]$*such that*$I_{1}\cup D_{1}=\left[K\right]$*and let some*$\left\{k\right\}\in D_{1}$, *if the channel satisfies the following conditions*

(A42) $$\begin{array}{c} \frac{h_{i+1}^{2}{\left(1+Q_{i+1}\right)}^{2}}{\rho_{i+1}^{2}}\leq 1-\rho_{i}^{2},\;\forall i\in I_{1}, \end{array}$$

(A43) $$\begin{array}{c} h_{k+1}\geq 1 \end{array}$$

(A44) $$\begin{array}{c} \prod_{\underset{j\neq \{k.k+1\}}{j=1}}^{K}\left(1+\frac{P_{j}}{1+Q_{j}}\right)\leq \frac{\prod_{j\in [K]}\frac{1}{2}\left(1+P_{j}+h_{j+1}^{2}P_{j+1}\right)}{\left(1+P_{k}+h_{k+1}^{2}P_{k+1}\right)}\;if\;D_{1}=\left[K\right]\\ \frac{\prod_{\underset{j\neq \{k.k+1\}}{j=1}}^{K}\left(1+\frac{P_{j}}{1+Q_{j}}\right)}{\prod_{j\in J_{2}}\left(1+c_{j}P_{j}\right)}\leq \frac{\prod_{j\in J_{1}}\left(1+P_{j}+h_{j+1}^{2}P_{j+1}\right)}{\left(1+P_{k}+h_{k+1}^{2}P_{k+1}\right)},\\ \forall J_{1}\subseteq D_{1},s.t.if\;i\in J_{1},\;then,\;i+1\notin J_{1}, \end{array}$$

(A45) $$\begin{array}{c} J_{2}=\left[K\right]\setminus \left\{i,i+1:i\in J_{1}\right\} \end{array}$$

*where*

$$Q_{i}=\left\{\begin{array}{cc} h_{i+1}^{2}P_{i+1} & :i\in I_{1}\\ 0 & :else \end{array}\right.$$

*the sum capacity is given by*

$$\begin{array}{c} C_{sum}=\frac{1}{2}\log\left(1+P_{k}+h_{k+1}^{2}P_{k+1}\right)+\sum_{\underset{j\neq \{k,k+1\}}{j=1}}^{K}\frac{1}{2}\log\left(1+\frac{P_{j}}{1+Q_{j}}\right) \end{array}$$

*where*$c_{i},\forall i\in \left[K\right]$*is defined as in Corollary A1*.

**Proof.** Use Theorem 3, to get the converse conditions (A42) and (A43) and use Corollary A1, to get the achievability conditions (A45). □

### Appendix E.2. Cascade GIC

We use the following channel model for cascade GIC

$$\begin{array}{cc} y_{k} & =x_{k}+h_{k+1}x_{k+1}+z_{k},\forall k\in \left\{1,2,\cdots ,K-1\right\}\\ y_{K} & =x_{K}+z_{K} \end{array}$$

**Result** **A3.**
*For the cascade GIC, satisfying the following conditions for some sets* $I_{1},D_{1}\subseteq \left[K\right]$ *and* $I_{1}\cup D_{1}\cup \left\{K\right\}=\left[K\right]$ *and* $\left\{K\right\}\notin I_{1},D_{1}$

(A46) $$\begin{array}{cc} \frac{h_{i+1}^{2}{\left(1+Q_{i+1}\right)}^{2}}{\rho_{i+1}^{2}} & \leq 1-\rho_{i}^{2},\;\forall i\in I_{1} \end{array}$$

(A47) $$\begin{array}{cc} h_{i+1}^{2}\left(1+Q_{i+1}\right) & \geq 1+P_{i},\forall i\in D_{1} \end{array}$$

*where*

$$Q_{i}=\left\{\begin{array}{cc} h_{i+1}^{2}P_{i+1} & :i\in I_{1}\\ 0 & :else \end{array}\right.$$

*the sum capacity is given by*

(A48) $$C_{sum}=\underset{i=1}{\sum^{K}}\frac{1}{2}\log\left[1+\frac{P_{i}}{1+Q_{i}}\right]$$

**Proof.** We get the result by taking $I\left(i\right)=\phi ,\;\forall i\in I_{1}$ and $I\left(i\right)=\left\{i+1\right\},\;\forall i\in D_{1}$ in Theorem 1. Noisy and mixed interference results in Reference [11] (Cor. 1), [11] (Thm. 3) can be obtained from this result. □

**Corollary** **A2.**
*For the cascade channel, if we treat interference as noise at receivers $i\in I_{1}$ and decode interference at receivers $i\in D_{1}=\left\{1,2,\cdots ,K-1\right\}\setminus I_{1}$, then the sum rates given by*

(A49) $$\begin{array}{cc} S & \leq \frac{1}{2}\sum_{j\in J_{1}}\log\left(1+P_{j}+h_{j+1}^{2}P_{j+1}\right)+\frac{1}{2}\sum_{j\in J_{2}}\log\left(1+e_{j}P_{j}\right)\\ \; & \forall J_{1}\subseteq D_{1},\;such\;that\;if\;\;i\in J_{1},\;then,\;i+1\notin J_{1}\\ \; & J_{2}=\left[K\right]\setminus \left\{i,i+1:i\in J_{1}\right\} \end{array}$$

*where, for all i=1,2,⋯,K−1*

$$e_{i}=\left\{\begin{array}{cc} \min\left\{h_{i}^{2},\frac{1}{1+h_{i+1}^{2}P_{i+1}}\right\} & :i-1\in D_{1},\;i\in I_{1}\\ \min\{h_{i}^{2},1\} & :i-1\in D_{1},\;i\in D_{1}\\ \frac{1}{1+h_{i+1}^{2}P_{i+1}} & :i-1\notin D_{1},\;i\in I_{1}\\ 1 & :i-1\notin D_{1},\;i\in D_{1} \end{array}\right.$$

*and $e_{K}=1$ are achievable.*

**Result** **A4.**
*For the cascade GIC, treating interference as noise at receivers* $i\in I_{1}$ *and decoding interference at receivers* $i\in D_{1}$ *(assuming* $\left\{K-1\right\}\in D_{1}$ *) is optimal if the channel satisfies the following conditions*

(A50) $$\begin{array}{c} \frac{h_{i+1}^{2}{\left(1+Q_{i+1}\right)}^{2}}{\rho_{i+1}^{2}}\leq 1-\rho_{i}^{2},\;\forall i\in I_{1}, \end{array}$$

(A51) $$\begin{array}{c} h_{K}\geq 1\\ \left(1+P_{K-1}+h_{K}^{2}P_{K}\right)\prod_{j=1}^{K-2}\left(1+\frac{P_{j}}{1+Q_{j}}\right)\leq \\ \;\;\prod_{j\in J_{1}}\left(1+P_{j}+h_{j+1}^{2}P_{j+1}\right)\prod_{j\in J_{2}}\left(1+e_{j}P_{j}\right),\\ \forall J_{1}\subseteq D_{1},\;such\;that\;if\;\;i\in J_{1},\;then,\;i+1\notin J_{1}, \end{array}$$

(A52) $$\begin{array}{c} J_{2}=\left[K\right]\setminus \left\{i,i+1:i\in J_{1}\right\} \end{array}$$

*where*

$$Q_{i}=\left\{\begin{array}{cc} h_{i+1}^{2}P_{i+1} & :i\in I_{1}\\ 0 & :else \end{array}\right.$$

*and the sum capacity is given by*

(A53) $$\begin{array}{c} C_{sum}=\frac{1}{2}\log\left(1+P_{K-1}+h_{K}^{2}P_{K}\right)+\sum_{j=1}^{K-2}\frac{1}{2}\log\left(1+\frac{P_{j}}{1+Q_{j}}\right) \end{array}$$

*where* $e_{i},\forall i\in \left[K\right]$ *is defined as in Corollary A2.*

**Proof.** Use Theorem 3 to get the converse conditions (A50) and (A51) and use Corollary A2 to get the achievability conditions (A52). Part of the strong interference result in Reference [11] (Cor. 2) can be obtained from this result. □

### Appendix E.3. Many-to-One GIC

Channel model for many-to-one IC is given by

(A54) $$\begin{array}{cc} y_{1} & =x_{1}+\sum_{j=2}^{K}h_{i}x_{i}+z_{i}\\ y_{i} & =x_{i}+z_{i},\;\forall i=2,3,\cdots ,K \end{array}$$

**Result** **A5.** *For a many-to-one channel, satisfying the following conditions*

(A55) $$\begin{array}{c} \sum_{j=k+1}^{K}h_{j}^{2}\leq 1\\ \prod_{i\in B-N}\left(1+P_{i}\right).\left(1+\underset{j=k+1}{\sum^{K}}h_{j}^{2}P_{j}+P_{1}\right)\leq \end{array}$$

(A56) $$\begin{array}{c} \;1+\sum_{i\in B-N}h_{i}^{2}P_{i}+P_{1},\forall N\subset B,N\neq B, \end{array}$$

*where*B = {2,3,⋯,k}, k∈{1,2,..,K}, *the sum capacity is given by then the sum capacity is given by*

(A57) $$\begin{array}{c} C_{sum}=\frac{1}{2}\log\left(1+\frac{P_{1}}{1+\sum_{j=k+1}^{K}h_{j}^{2}P_{j}}\right)+\sum_{i=2}^{K}\frac{1}{2}\log\left(1+P_{i}\right) \end{array}$$

**Proof.** From Theorem 1, taking I(1)={k+1,⋯,K}, we get the required sum capacity if the channel satisfies the conditions (A55) and also

$$\sum_{j=k+1}^{K}\frac{h_{j}^{2}}{\rho_{j}^{2}}\leq 1-\rho_{1}^{2}$$

for some $\rho_{i}\in \left[0,1\right],\;i=1,2,\cdots ,K$. Choose $\rho_{1}=0$ and $\rho_{j}=1\;\forall j=k+1,\cdots ,K$ to get the condition (A55). Reference [13] (Thm. 4) can be obtained from this result. □

**Result** **A6.**
*For the K-user Gaussian many-to-one IC satisfying the following channel conditions:*

$$\begin{array}{c} \prod_{i\in N}\left(1+P_{i}\right)\left(1+P_{1}+\underset{i=k+1}{\sum^{K}}h_{i}^{2}P_{i}+\sum_{i\in B-N}h_{i}^{2}P_{i}\right) \end{array}$$

(A58) $$\begin{array}{c} \geq \prod_{i=2}^{k-1}\left(1+P_{i}\right)\left(1+P_{1}+\sum_{j=k}^{K}h_{j}^{2}P_{j}\right)\\ \forall N\subseteq B,N\neq \{2,3,..,k-1\}\;and\;B=\{2,3,...k\} \end{array}$$

(A59) $$\begin{array}{c} \underset{i=k+1}{\sum^{K}}h_{i}^{2}\leq 1-\rho^{2},\;\;\rho h_{k}=1+\underset{i=k+1}{\sum^{K}}h_{i}^{2}P_{i} \end{array}$$

*the sum capacity is given by*

(A60) $$\begin{array}{c} C_{sum}=\underset{\underset{i\neq k}{i=2}}{\sum^{K}}\frac{1}{2}\log\left(1+P_{i}\right)+\frac{1}{2}\log\left(1+\frac{P_{1}+h_{k}^{2}P_{k}}{1+\underset{i=k+1}{\sum^{K}}h_{i}^{2}P_{i}}\right) \end{array}$$

**Proof.** (Converse) From Theorem 3, taking G(i)={k+1,⋯,K}, we get the required outer bound when (A59) is satisfied. (Achievability) Using Theorem 2 with I(i)={k+1,⋯,K}, we get the following achievable sum rates

(A61) $$\begin{array}{c} S\leq \frac{1}{2}\underset{i=k+1}{\sum^{K}}\log\left(1+P_{i}\right)+\frac{1}{2}\sum_{i\in M}\log\left(1+m_{i}P_{i}\right)+\frac{1}{2}\;\log\left(1+\frac{P_{1}+\sum_{i\in B-M}h_{i}^{2}P_{i}}{1+\underset{i=k+1}{\sum^{K}}h_{i}^{2}P_{i}}\right),\forall M\subseteq B. \end{array}$$

where B={2,3,⋯,k} and $m_{i}=\min\left\{1,\frac{h_{i}^{2}}{1+\underset{j=k+1}{\sum^{K}}h_{j}^{2}P_{j}}\right\}$. Among these sum rates, we want the sum rate with M=B∖{k} and $m_{i}=1$, ∀i∈B∖{k} to be dominant. We get the conditions (A58), for the sum rate with M=B∖{k} to be dominant assuming $m_{i}=1,\forall i\in \left\{2,3,\cdots ,k\right\}$. Given the converse conditions (A59) and (A58), conditions with $m_{1}=\frac{h_{i}^{2}}{1+\underset{j=k+1}{\sum^{K}}h_{j}^{2}P_{j}}$ are always redundant. Reference [13] (Thm. 5) can be obtained from this result. □

### Appendix E.4. One-to-Many GIC

We use the following model for one-to-many channel

(A62) $$\begin{array}{cc} y_{i1} & =x_{i}+h_{i}x_{K}+z_{i}\;i=2,\cdots ,K-1\\ y_{K} & =x_{K}+z_{K} \end{array}$$

**Result** **A7.**
*For the K user Gaussian one-to-many channel satisfying the following conditions*

(A63) $$\begin{array}{c} 1+P_{i}\leq {\left|h_{i}\right|}^{2},1\leq i\leq k, \end{array}$$

(A64) $$\begin{array}{c} \underset{j=k+1}{\sum^{K-1}}\frac{|h_{j}{|}^{2}P_{K}+{\left|h_{j}\right|}^{2}}{|h_{j}{|}^{2}P_{K}+1}\leq 1, \end{array}$$

*then the sum capacity is given by*

$$\begin{array}{c} C_{sum}=\frac{1}{2}\;\underset{i=1}{\sum^{k}}\log\left(1+P_{i}\right)+\frac{1}{2}\;\log\left(1+P_{K}\right)+\frac{1}{2}\;\underset{j=k+1}{\sum^{K-1}}\log\left(1+\frac{P_{j}}{1+|h_{j}{|}^{2}P_{K}}\right) \end{array}$$

**Proof.** From Theorem 1, taking I(i)=ϕ,∀i={1,2,⋯,k} and I(i)={K},∀i={k+1,⋯,K−1}, we get the required sum capacity under the conditions (A63) and

$$\sum_{j=k+1}^{K-1}\frac{h_{j}^{2}}{1+h_{j}^{2}P_{K}-\rho_{j}^{2}}\leq \frac{1}{1+{\left(\frac{1}{\rho_{K}}\right)}^{2}}$$

Choosing $\rho_{K}=1$, $\rho_{j}=0,\forall j=k+1,\cdots ,K-1$, we get (A64). Reference [13] (Thm. 6) can be obtained from this result. □

**Result** **A8.** *For the K-user Gaussian one-to-many IC satisfying the following conditions*:

(A65) $$\begin{array}{cc} 1\leq h_{l}^{2} & \leq 1+P_{l} \end{array}$$

(A66) $$\begin{array}{cc} \frac{h_{l}^{2}}{1+P_{l}} & \leq \frac{h_{i}^{2}}{1+P_{i}},\;1\leq i\leq K-1,\;i\neq l \end{array}$$

*for any*l∈{1,2,⋯,K−1}, *the sum capacity is*

(A67) $$C_{sum}=\frac{1}{2}\underset{j=1,j\neq l}{\sum^{K-1}}\log\left(1+P_{j}\right)+\frac{1}{2}\log\left(1+P_{l}+h_{l}^{2}P_{K}\right)$$

**Proof.** From Theorem 3, with G(i)={K},∀i={1,2,⋯,K−1}, sum rate is upper bounded by (A67). From Theorem 2, we get the following achievable sum rates

$$\begin{array}{cc} S & \leq \underset{j=1}{\sum^{K}}\frac{1}{2}\;\log\left(1+P_{j}\right),\\ S & \leq \underset{j\neq i}{\underset{j=1}{\sum^{K-1}}}\frac{1}{2}\;\log\left(1+P_{j}\right)+\frac{1}{2}\;\log\left(1+P_{i}+h_{i}^{2}P_{K}\right),\;\forall \;1\leq i\leq K-1 \end{array}$$

For the required sum rate to be dominant among all the achievable rates, channel should satisfy (A65) and (A66). Reference [13] (Thm. 7) can be obtained from this result. □

## Appendix F. Model Parameters Used in the Numerical Results

For channel fading, we use the Erceg model [21]. The path loss function is given by

$$PL=20\log_{10}\left(\frac{4\pi d_{0}}{\lambda }\right)+10\gamma \log_{10}\left(\frac{d}{d_{0}}\right)+s,$$

where $d_{0}$ is some close-in distance, λ is wavelength, γ is path loss exponent, *s* is shadow fading component. γ is characterized as follows

$$\gamma =\left(a-bh_{b}+\frac{c}{h_{b}}\right)+x\sigma_{\gamma },$$

where $h_{b}$ is antenna height, x∼N(0,1), $a,b,c,\sigma_{\gamma }$ are constants which depends on the terrain type.

The shadow fading component *s* is given by

$$s=y(\mu_{\sigma }+z\sigma_{\sigma }),$$

where y,z∼N(0,1), $\mu_{\sigma },\sigma_{\sigma }$ are constants which depends on the terrain type. We consider two terrain categories, hilly/light tree density (terrain type 1) and hilly/moderate-to-heavy tree density (terrain type 2). The model parameters for the two terrain categories are given in the Table A5.

**Table A5:** Numerical values of model parameters.

Model Parameter	Terrain Category	
	Hilly/Light Tree Density	Hilly/Moderate-to-Heavy Tree Density
a	4.0	4.6
b (in m${}^{-1}$)	0.0065	0.0075
c (in m)	17.1	12.6
$\sigma_{\gamma }$	0.75	0.57
$\mu_{\sigma }$	9.6	10.6
$\sigma_{\sigma }$	3.0	2.3
